# Expression of GPR34 in microglia remains stable in human Alzheimer’s disease

**DOI:** 10.1007/s00401-026-03035-0

**Published:** 2026-06-15

**Authors:** Sophie Seiffer, Jonas Rotter, Jana Brendler, Albert Ricken, Zoe Detzer, Max Braune, Torsten Schöneberg, Angela Schulz, Karsten Winter, Ingo Bechmann

**Affiliations:** 1 Institute of Anatomy, Faculty of Medicine, Leipzig University, Liebigstraße 13, 04103 Leipzig, Germany; 2 Paul Flechsig Institute of Neuropathology, University Hospital Leipzig, Leipzig, Germany; 3 Rudolf Schönheimer Institute of Biochemistry, Faculty of Medicine, Leipzig University, Leipzig, Germany; 4 Department of Biochemistry, School of Medicine, University of Global Health Equity (UGHE), Kigali, Rwanda; 5 LeiCeM - Leipzig Center of Metabolism, Leipzig University, Leipzig, Germany

**Keywords:** Neuropathology, Neurodegenerative disease, Glia, Neurofibrillary tangles, Amyloid beta plaques

## Abstract

Microglia are the resident immune cells of the human central nervous system and play key roles in development, homeostasis, and disease. These functions are mediated by a broad repertoire of cell-surface receptors, including G protein-coupled receptors such as the ADP receptor P2Y12 and GPR34, a receptor for lysophosphatidylserine. While GPR34 deficiency has been linked to impaired microglial phagocytosis, its regulation in relation to amyloid-β (Aβ) and tau pathology in Alzheimer’s disease (AD) remains unclear. We performed a quantitative analysis of microglial density, morphology, and GPR34 expression in the medial temporal lobe cortex (MTLC) of elderly human body and tissue donors across the AD spectrum. Using fluorescence in situ hybridization and immunolabeling, we analyzed 187,670 microglial cells and correlated microglial parameters with the severity and spatial proximity of Aβ plaques and tau inclusions. In parallel, we analyzed human single-nucleus RNA sequencing data from 236,002 cells to assess GPR34 expression across microglial subtypes, brain regions, and neuropathological stages. Microglial density and overall morphology in the MTLC were largely preserved, independent of local Aβ or hyperphosphorylated tau burdens. Apart from a moderate shortening of microglial processes in the immediate vicinity of Aβ plaques, no consistent pathology-associated morphological changes were detected. GPR34 expression showed pronounced cell-to-cell variability and differed across microglial subtypes and brain regions, but neither expression intensity nor the proportion of GPR34-positive microglia correlated consistently with Braak stage or Thal phase. These findings suggest that GPR34 regulation in human microglia is highly context-dependent and shaped by regional and cellular heterogeneity rather than AD-associated pathology alone.

**Supplementary Information:**

The online version contains supplementary material available at 10.1007/s00401-026-03035-0.

## Introduction

Alzheimer’s disease (AD) is a progressive neurodegenerative disorder characterized by synaptic dysfunction, neuronal cell loss, and cerebral atrophy, leading to memory loss and cognitive decline [110]. Histopathologically, AD is characterized by the accumulation of hyperphosphorylated tau (p-tau; AT8-positive) protein/tangles in degenerating neurons and the extracellular deposition of aggregated amyloid-β (Aβ) peptides [5].

Mice do not spontaneously develop these core pathological hallmarks of human AD, including progressive neuronal loss, extensive Aβ deposition, and AT8-positive neurofibrillary tangles (NFTs), under physiological conditions. Consequently, most studies rely on genetically engineered mouse models that overexpress human Aβ and/or p-tau by introducing human transgenes and/or mutations in genes such as APP, PSEN1, or MAPT. In genetically engineered mouse models of AD, microglia accumulate around the artificial protein deposits [19, 60, 113, 129]. Transcriptomic profiling of these microglia reveals an upregulation of genes characteristic of so-called “disease-associated microglia” (DAM), including mediators of pro-inflammatory signaling, cytoskeletal remodeling, and phagocytosis [63, 65]. Furthermore, genes typically expressed by microglia under homeostatic conditions, including the G protein-coupled receptors (GPCR) P2RY12, P2RY13, CX3CR1, and GPR34, are strongly regulated. Of particular interest is the regulation of GPR34, as its expression in microglia is markedly downregulated in aging mouse and human brains, with aging as the strongest human risk factor for AD [39, 51, 65, 90, 124, 128].

GPR34 is a core signature gene in both mouse and human microglia, owing to its highly selective expression in microglia relative to other brain cell types [14, 24, 45, 51]. It encodes a GPCR of the rhodopsin class, within the P2RY12-like subgroup, whose physiological ligand and exact function in vivo remain incompletely defined [39, 108]. Lysophosphatidylserine, a lipid released during apoptosis, has been proposed as a candidate ligand; however, conflicting findings have left its role unresolved [8, 67, 94, 102, 117].

In prior studies, we have investigated the function of GPR34 using knockout (KO) mice [56, 67, 97, 107]. In the absence of GPR34, microglia exhibit elevated cytokine levels and impaired phagocytosis [67, 97]. Consistent with these alterations, GPR34-deficient mice show higher pathogen loads in multiple organs, including the central nervous system (CNS), and increased susceptibility to systemic infections [67]. The effects of GPR34-KO on the morphology of microglia are less clear, with one study reporting a shift toward an amoeboid phenotype, whereas another found no measurable changes in the branching of microglial processes [70, 97]. Despite the uncertainty about its endogenous ligand, biochemical studies have provided insights into GPR34 downstream signaling. By coupling to Gi/Go proteins, GPR34 inhibits adenylyl cyclase and activates MAPK/ERK and PI3K/Akt signaling, leading to the induction of transcription factors such as c-FOS, c-JUN, and NF-κB [7, 107, 108, 134]. Importantly, ERK signaling has been implicated in AD [26, 34]. In particular, it appears to be upregulated by Aβ and to correlate with cognitive decline in affected individuals [64, 99].

Based on these and other findings, transgenic AD models carrying an additional GPR34-KO were generated to investigate the role of GPR34 in AD pathology. In APP/PS1 mice, GPR34 deletion improved learning and memory and reduced neuroinflammation, suggesting that increased GPR34 activity may contribute to cognitive impairment and inflammatory responses in AD [71]. Consistent with this hypothesis, Zhou et al. showed that GPR34 deletion in 5xFAD mice, a model of age-dependent Aβ accumulation, reduces microglia-mediated inflammation, improves microglial phagocytosis of Aβ, and lowers both Aβ and p-tau levels in the brain [132]. Similarly, exposure of 5xFAD mice to a selective GPR34 antagonist reduced pro-inflammatory gene expression and Aβ deposition, supporting the hypothesis that GPR34 signaling contributes to AD pathogenesis and may represent a therapeutic target. Interestingly, GPR34 deficiency enhanced microglial phagocytosis in 5xFAD mice [132], but impaired it in mice without AD transgenes [97], suggesting that the impact of GPR34 signaling on microglial phagocytosis may be influenced by the respective microenvironment.

However, the translational validity of these models is limited in several respects. First, no transgenic mouse model developed to date recapitulates the full spectrum of human AD pathology [73, 123]. Amyloid precursor protein (APP)-based mouse models typically depend on supraphysiological expression of human APP, resulting in large, predominantly Aβ40-enriched plaques that differ structurally and biochemically from the mainly Aβ42-containing plaques in humans, and they also fail to develop NFTs or widespread neurodegeneration unless mutant tau alleles are additionally introduced [84, 103]. Second, neuroinflammatory and glial responses in AD mouse models diverge fundamentally from those in the human disease. Species-specific differences in microglial distribution, transcriptional programs, and immune responsiveness limit cross-species comparability, and are further confounded by the exposure of murine microglia to ectopically expressed or structurally altered human proteins [9, 17, 36, 41, 84, 112]. Finally, these models insufficiently capture aging, as mice are typically studied between 3 and 12 months of age, corresponding to less than 50 years in humans [40, 46]. Recent studies show that DAM gene expression patterns differ substantially between microglia from AD patients and those from APP-based mouse models, underscoring the need to validate mouse-derived findings in human tissue [3, 28, 89].

The aim of the present study was to investigate whether phenotypic and GPR34 transcriptional changes observed in transgenic mouse models of AD are also present in humans. To this end, we examined microglia in the medial temporal lobe cortex (MTLC), which includes the transentorhinal cortex (TEC; Brodmann area 35), the earliest site of tau pathology and a key region in the development of AD [16, 22, 72, 127]. Instead of only assessing the mere presence of pathological changes in individual brain regions (used for Braak staging), we also quantified the density and spatial distribution of Aβ- and AT8-positive deposits to determine their regional burden more accurately. We then analyzed how microglial alterations relate to the presence and proximity of Aβ- or AT8-positive deposits, focusing on GPR34 expression and cell morphology. In ionized calcium-binding adaptor molecule 1 (Iba1)-stained microglia, morphological features and the presence of GPR34 mRNA were analyzed and correlated with Aβ- or AT8-labeled deposits using a combined fluorescent in situ hybridization (FISH) and immunolabeling approach. To substantiate our tissue findings and further characterize GPR34 expression patterns across microglial subtypes in human microglia, we additionally analyzed single-nucleus RNA sequencing (snRNA-seq) data from multiple brain regions, and correlated GPR34 expression with Braak stages (refers to tau staging) and Thal phases (refers to amyloid staging).

Here, we provide a single-cell characterization of GPR34 expression in microglia of the aging human brain, analyzed in relation to microglial phenotype and to the presence, extent, and spatial proximity of Aβ and p-tau deposits, as well as across microglial subtypes and brain regions using large-scale snRNA-seq datasets. We observed only minor alterations in GPR34 expression and microglial morphology associated with AD-related deposits, and no consistent directional changes in GPR34 expression across disease progression. Our findings reveal important differences from existing mouse models, shedding new light on the role of microglia in human AD brains.

## Materials and methods

### Subjects and case selection

**Fig. 1:**
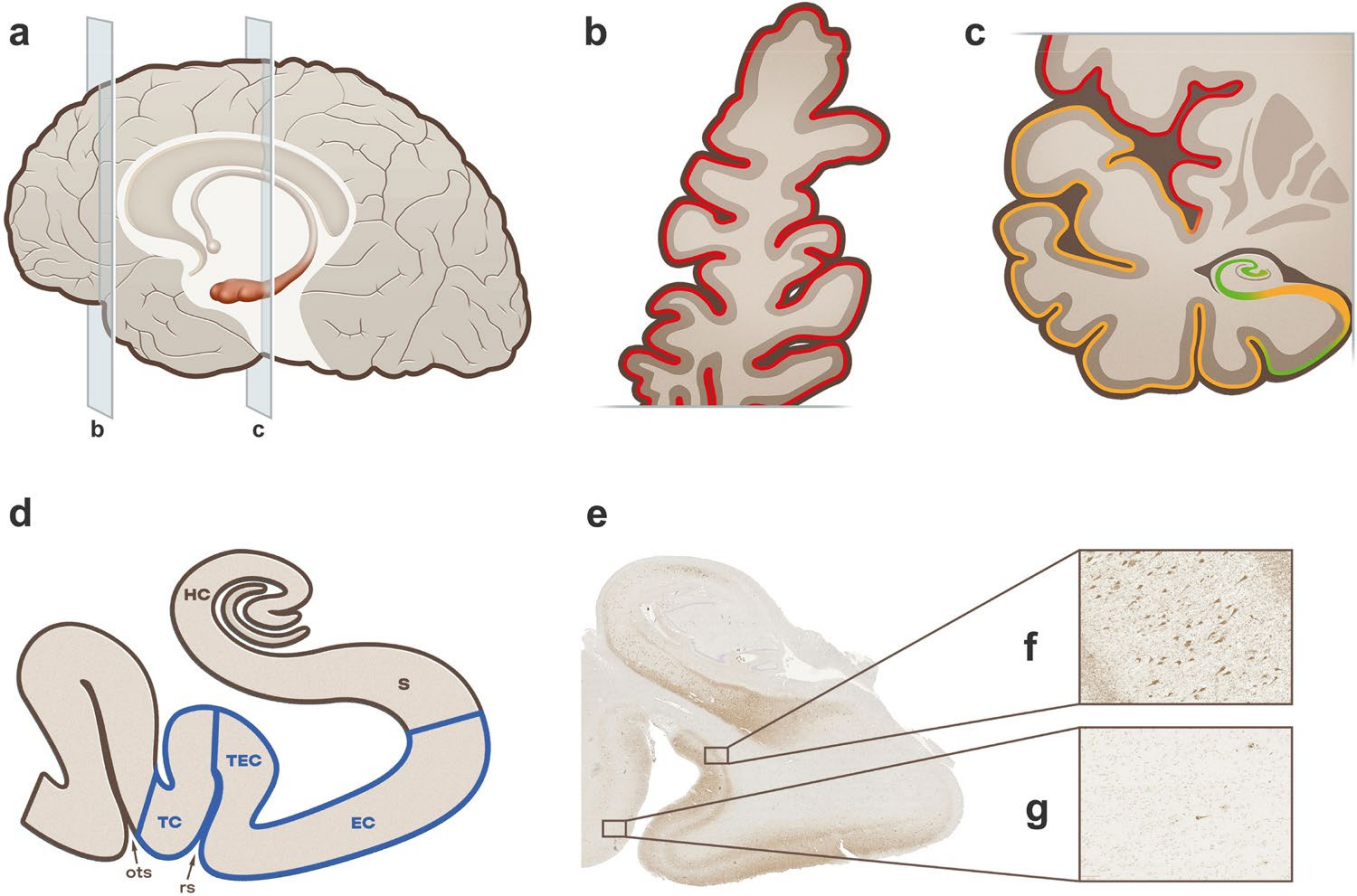
Anatomical regions examined and Braak staging of donor brains. **a** Midsagittal view of the human brain showing the coronal planes used for sampling of the prefrontal cortex (PFC) and medial temporal lobe cortex (MTLC). The hippocampal formation is highlighted in red and projected onto the midsagittal plane to indicate the coronal plane of MTLC sampling. **b** and **c** Schematic coronal sections of the PFC (b) and MTLC (c), illustrating the hierarchical progression of tau pathology in AD according to Braak and Braak [21]. Regions are color-coded by the Braak stage at which they are first affected: green for stages I–II, yellow for stages III–IV, and red for stages V–VI. **d** Enlargement of the cortical area of a coronal MTLC section illustrating anatomical subdivisions. Regions included in the analyses are outlined in blue: EC = entorhinal cortex, including the transentorhinal region (TEC); TC = temporal cortex. Adjacent anatomical landmarks are outlined in black: HC = hippocampus, S = subiculum, rs = rhinal sulcus, ots = occipitotemporal sulcus. **e** Whole-slide scan of an MTLC section from a representative case (Braak stage III), stained for hyperphosphorylated tau (p-tau) with anti-AT8 and DAB immunohistochemistry. **f** and **g** Higher-magnification views of EC (f) and TC (g) from the same section, showing regional differences in AT8 staining. This figure was created using Adobe Photoshop v26.0

MTLC samples were obtained from human brains donated to the Institute of Anatomy or examined at the Paul Flechsig Institute of Neuropathology, University of Leipzig. All donors had provided written informed consent for the use of their organs in scientific research. Sample acquisition was approved by the Ethics Committee of the University of Leipzig (approval no. 129-21-ek) and complied with institutional and national regulations. In line with previous studies, including our own work on post-mortem tissue suitability for mRNA detection by FISH, samples were excluded if the post-mortem interval (PMI) to fixation exceeded 48 h, or 74 h under continuous cooling [109]. Additional exclusion criteria included CNS pathologies (cerebral hemorrhages, brain tumors, and ischemic injuries) and systemic infectious conditions such as sepsis, if deemed likely to affect the brain tissue integrity or neuropathological assessment. Since detailed medical histories were not consistently available, inclusion was based on the available clinical information and neuropathological examination of brain tissue. On this basis, one donor (Case #1) with a documented diagnosis of septic shock was retained, as the available clinical information indicated that sepsis was not the primary cause of death, and the analyzed brain tissue showed no evidence of sepsis-associated CNS pathology. As tau pathology is the most robust morphological correlate of clinical dementia severity, samples were AT8-immunolabeled to detect NFTs and staged according to Braak criteria [20, 75, 85, 86] (Fig. 1). Twenty cases spanning Braak stages 0–VI were selected for analysis (n = 6 for stages 0–II, 7 for III–IV, 7 for V–VI). Donors included both sexes, aged 54−97 years. Comprehensive donor information is summarized in Table 1.

**Table 1:** Summary of demographic and clinical data from the 20 donors of post-mortem brains included in this study

Case ID	Age (y)	Sex	BMI	PMI (h)	Braak	ICD-10
1	55	m	29.2	72	0	K72.0, R57.2, C22.0, K70.3, K76.6, J18.0
2	72	m	31.1	48	2	I50.0, I25.2, I25.1, I70.9, E11.9, I10, E78.5
3	86	f	20.8	34	4	N17.9, E86.0, N18.0, E11.9, D63.8, I48.1
4	71	m	44.2	72	1	K72.0, R57.2, C22.0, K70.3, I51.7, I25.2
5	60	m	33.3	48	2	R57.2, I50.0, K76.6, I84.3, K70.3, C67.9, I48.1, Z92.1, E11.9, I10, N18.3
6	54	f	47.2	48	2	K72.0, C78.7, C50.9, Z51.1, Z51.0
7	72	m	26.9	72	4	R57.2, I26.9, J18.9, I69.3
8	70	m	25.1	72	4	R57.2, I50.0, K31.819, K92.2, I25.1, I25.2, I23.3, I82.2, I26.0
9	86	f	25.5	52	5	J25.1, J11, J18.9
10	63	m	24.7	24	2	R57.0, K31.82, I25.10, I23.4, Z95.812, Z79.01, I82.9, I26.9
11	87	m	21.9	48	4	R57.2, I50.0, J18.9, G30.9, I25.1, Z95.5, I25.2, C61
12	80	f	32.9	32	4	J99.0, M05.9, I42.9, I48.0, I10, I25.9
13	81	m	16.0	16	5	C34.9, N18.3
14	86	f	24.7	17	5	I50.1, N18.0, F03
15	88	m	15.2	14	3	C25.0, E11.9
16	83	m	27.0	48	4	I50.0, J69.0, G30.9, I67.7, G25.8, I10, I70.9, I25.1, I51.7, I51.8
17	78	m	24.3	19	5	J96.0, E67.9, C61
18	87	f	33.8	27	6	J96.0, I50.9, I48, E11, N17, E87.5
19	86	m	28.7	18	6	J15.9, J98.1, I42.0, I69.3, F01.3, I48.0
20	97	f	19.5	16	6	I50.9, I48.2, I10.9, R64

CASE ID = case Identification number, AGE (y) = age at death in years (y), SEX = male (m) or female (f), BMI = body mass index, PMI (h) = post-mortem interval in hours (h), BRAAK = Braak stage, ICD-10 = international statistical classification of diseases and related health problems (10th Revision) codes recorded on the death certificate

### Classification of cases based on Aβ and tau pathology

**Fig. 2:**
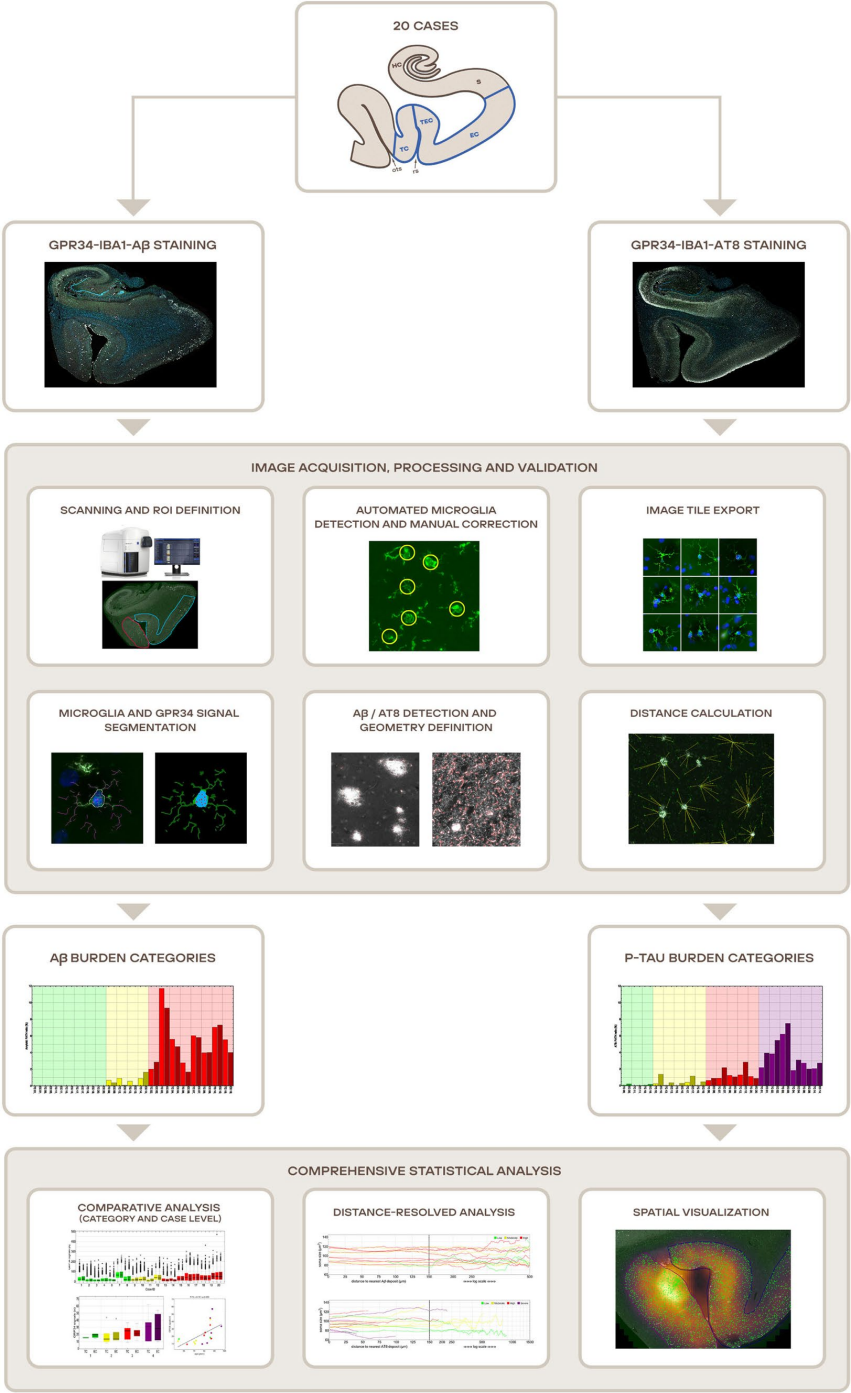
Workflow for spatial single-cell analysis of microglia in relation to Aβ- and AT8-positive deposits. Overview of the experimental and analytical pipeline. Post-mortem human brain tissue from 20 cases (Braak stages 0–VI) was examined for GPR34 expression, Iba1-positive microglia, and the presence of Aβ or p-tau (AT8-positive) deposits using combined fluorescence in situ hybridization and immunofluorescence staining. Whole-slide images were acquired, and regions of interest in the entorhinal cortex (EC) and temporal cortex (TC) were manually outlined. Microglia were identified by automated detection with subsequent manual quality control, and GPR34 signals were quantified at single-cell resolution. In parallel, Aβ- and AT8-positive deposits were detected, and distances from each microglial cell to the nearest deposits were computed. Aβ and p-tau burdens were quantified and used to assign cases to three (Aβ) and four (p-tau) burden categories. Subsequently, GPR34 signal counts, microglial morphology (soma size and proximal process length), and microglial density were compared across burden categories and between cases, and further analyzed in relation to the distance from Aβ- and p-tau deposits. Results were plotted and visualized as spatial heatmaps in EC and TC. One case was excluded from the study due to the absence of an Iba1-positive microglia reaction. This figure was created using Adobe Photoshop v26.0

To examine microglial changes in relation to pathological burden, cases were classified by the regional extent of Aβ- and AT8-positive deposits in the entorhinal cortex (EC) and temporal cortex (TC). Deposit burden was quantified as the percentage of tissue area occupied by Aβ- or AT8-positive deposits (see workflow Fig. 2). Cases were categorized according to Aβ burden in the EC and TC: low (< 0.208%), moderate (0.208–2.698%), and high (> 2.698%). Tau pathology was classified into four categories based on p-tau deposit abundance and anatomical progression within the EC and TC: low (≤ 0.206% in EC and TC), moderate (TC ≤ 0.405%, EC > 0.206%), high (≥ 0.638% in both regions), and severe (≥ 1.834% in both regions). Thresholds reflected the spatio-temporal sequence of tau pathology, which initiates in the EC and gradually spreads into adjacent neocortical areas [20]. Categories were validated by visual cross-comparison to ensure regional consistency.

### Tissue sampling, preparation, and fixation

MTLC samples were dissected from coronal slices encompassing the hippocampus, EC, TEC, and TC. For Braak staging, additional prefrontal cortex (PFC) sections were collected (Fig. 1). Samples designated for immunohistochemistry (IHC), or combined FISH and immunofluorescence (FISH-IF) were immediately fixed in dH_2_O with 4% formaldehyde (Roti®Histofix, Carl Roth, Karlsruhe, Germany) for 24 h (FISH-IF) or 14 days (IHC), dehydrated in graded ethanol and xylene, and embedded in paraffin. Ten-µm-thick serial sections were cut from the paraffin-embedded samples and mounted on glass slides. Selected sections were stained with haematoxylin and eosin (HE) and Nissl for anatomical reference. Sections adjacent to those best capturing the region of interest were used for staining and analysis.

### Immunohistochemistry (IHC)

Paraffin sections were rehydrated in xylene and ethanol, followed by heat-induced antigen retrieval in citrate buffer (pH 6.0) for 20 min at 95 °C. Sections were rinsed in 0.02 M phosphate-buffered saline (PBS, pH 7.4), and endogenous peroxidase was quenched with 3% hydrogen peroxide for 30 min at room temperature (RT). Sections were washed in PBS with 0.3% Triton X-100 (PBS-T) and blocked with 5% normal goat serum (NGS) in PBS-T for at least 1 h at RT. Primary antibodies used were a monoclonal antibody against phospho-tau (AT8, Ser202, Thr205; 1:500, Thermo Fisher Scientific, Cat. No. MN1020) and a monoclonal anti-β-amyloid antibody (1:5,000, Sigma-Aldrich, Cat. No. A8354). Antibodies were diluted in PBS-T with 1% NGS and 0.5% bovine serum albumin and applied overnight at 4 °C. After washing, sections were incubated with biotinylated secondary antibody (goat anti-mouse IgG, 1:200) for 2 h at RT, followed by signal amplification using ExtrAvidin-peroxidase (1:200, 1 h, RT) and visualization with DAB. Sections were counterstained with Mayer’s hemalum, dehydrated, cleared in xylene, and cover-slipped.

### Fluorescence in situ hybridization and immunofluorescence (FISH-IF)

FISH-IF was performed on deparaffinized sections using the RNAscope™ Multiplex Fluorescent Detection Kit v2 (ACD, Cat No. 323100) with Opal 570 dye (Akoya Biosciences, Cat. No. SKU FP1488001KT). Sections were baked at 60 °C for 1 h, deparaffinized and rehydrated in xylene and ethanol. Endogenous peroxidase activity was blocked by incubation in hydrogen peroxide (10 min, RT), followed by boiling in RNAscope 1X Target Retrieval Reagent (35 min) to retrieve antigens. Sections were incubated with RNAscope™ Hs-GPR34 probe (1:50, ACD, Cat No. 521021; NM_005300.3; bp position 210–1305) for 2 h at 40 °C using the HybEZ™ II manual hybridization system (ACD). Signal amplification and Opal 570 labeling were performed according to the manufacturer’s instructions (1:750 dilution). After RNA labeling, sections were blocked in PBS-T with 3% NGS and co-immunolabeled with monoclonal recombinant Iba1 antibody (1:5,000; Synaptic Systems, Cat. No. 234 308) in combination with either AT8 or Aβ antibodies as described above. Secondary antibodies were Alexa Fluor 647 goat anti-guinea pig (Iba1, 1:250) and Alexa Fluor 488 goat anti-mouse (AT8/Aβ, 1:250). Nuclei were counterstained with DAPI for 30 s at RT and briefly immersed in TrueBlack to suppress tissue autofluorescence. Sections were cover-slipped with Dako fluorescence mounting medium.

### Image acquisition

Slides were digitized using an Axioscan 7 scanner (Carl Zeiss Microscopy GmbH, Jena, Germany) operated with ZEN microscope software (v3.7). Bright-field scans for IHC, HE, and Nissl were acquired at 20×. FISH-IF slides were imaged at 40× (pixel (px) size: 0.172 µm) as 11-layer z-stacks (0.5 µm inter-slice). Four fluorescence channels were recorded: DAPI, AF647 (Iba1), AF488 (Aβ or AT8) and Cy3 (GRP34 mRNA). To generate projection in a single plane, an extended depth of focus operation was applied to each image stack. Detailed image acquisition parameters are provided in Supplementary Table S1.

### Spatial in situ analysis

Regions of interest (ROIs) for EC and TC were manually annotated on fluorescence images based on anatomical landmarks in corresponding HE- and Nissl-stained sections (Fig. 1d). Cell detection was performed in QuPath (v0.5.1) using the StarDist plugin (v0.5.0) for deep learning-based nucleus detection in the DAPI channel [10, 106]. Iba1-positive microglia were identified by AF647 intensity thresholds (median >375, standard deviation >100) and DAPI overlap. False positives were excluded using an additional AF488 threshold and a nucleus–cytoplasm overlap criterion (detailed parameters in Supplementary Table S2). All automated detections were manually curated to remove non-microglial cells and add missing microglia. A QuPath object classifier was trained on manually validated detections (29,435 positive, 591,153 negative), achieving 97% training accuracy and 86% concordance after manual review. Image tiles (401x401 px) centered on each validated microglial cell were exported. Aβ deposits were detected using threshold-based segmentation with Gaussian filtering and percentile normalization (Gaussian filter 1, normalize percentiles [1, 99], threshold 0.15, pixel size factor 2.0). AT8-positive deposits were detected with a pixel classifier trained on annotated datasets covering diverse pathology stages. All detections were manually corrected and exported in GeoJSON format.

### Image processing and quantification

Image processing was performed in Mathematica (v14, Wolfram Research Inc., Champaign, IL, USA). Microglial somata were segmented in the AF647 channel by Otsu thresholding and morphological operations [91]. Processes were segmented using a ridge filter, local adaptive binarization, and thinning. Processes within 75 px of a soma were assigned to the corresponding cell, and skeletonization enabled measurement of total proximal process length (µm). Local microglial density (within a 150 µm radius) and distances to nearest Aβ and p-tau deposits were calculated for each cell. GPR34 mRNA signals were segmented in the Cy3 channel using local adaptive thresholding. Signal quantification followed the manufacturer’s protocol (“Guideline on how to quantify RNAscope^®^ Fluorescent Assay Results”) [2], with background-corrected cumulative intensity divided by average single-dot intensity. All parameters were quantified at single-cell resolution, with regional values (EC, TC) represented by medians. Aβ and p-tau burdens were defined as percentage of tissue area occupied within ROIs, and regional microglial density as total number of microglia per ROI area (cells/mm^2^). For detailed image processing and quantification parameters, refer to Supplementary Table S3.

### Distance analysis

The distance from each microglia to the nearest Aβ- or AT8-positive deposit was calculated. Microglia were binned by distance (1 µm intervals), and median values for soma size, proximal process length, density, and GPR34 counts were computed for each interval. Profiles were smoothed with a 25-point mean filter and truncated to reduce edge effects. Spatial distributions were visualized in color-coded histograms.

### Single-nucleus RNA-seq analysis

Preprocessed snRNA-seq data from the SEA-AD study were analyzed, focusing on 236,002 microglial nuclei from 84 donors across multiple brain regions, including middle temporal gyrus (MTG), superior temporal gyrus (STG), primary visual cortex (V1C), medial entorhinal cortex (MEC), lateral entorhinal cortex (LEC), hippocampal formation (HIP), inferior temporal gyrus (ITG), angular gyrus (AnG), frontoinsular cortex (FI), and dorsolateral frontal cortex (DFC) [44]. Comprehensive information about the SEA-AD cohort is available at https://brain-map.org/consortia/sea-ad.

**Fig. 3:**
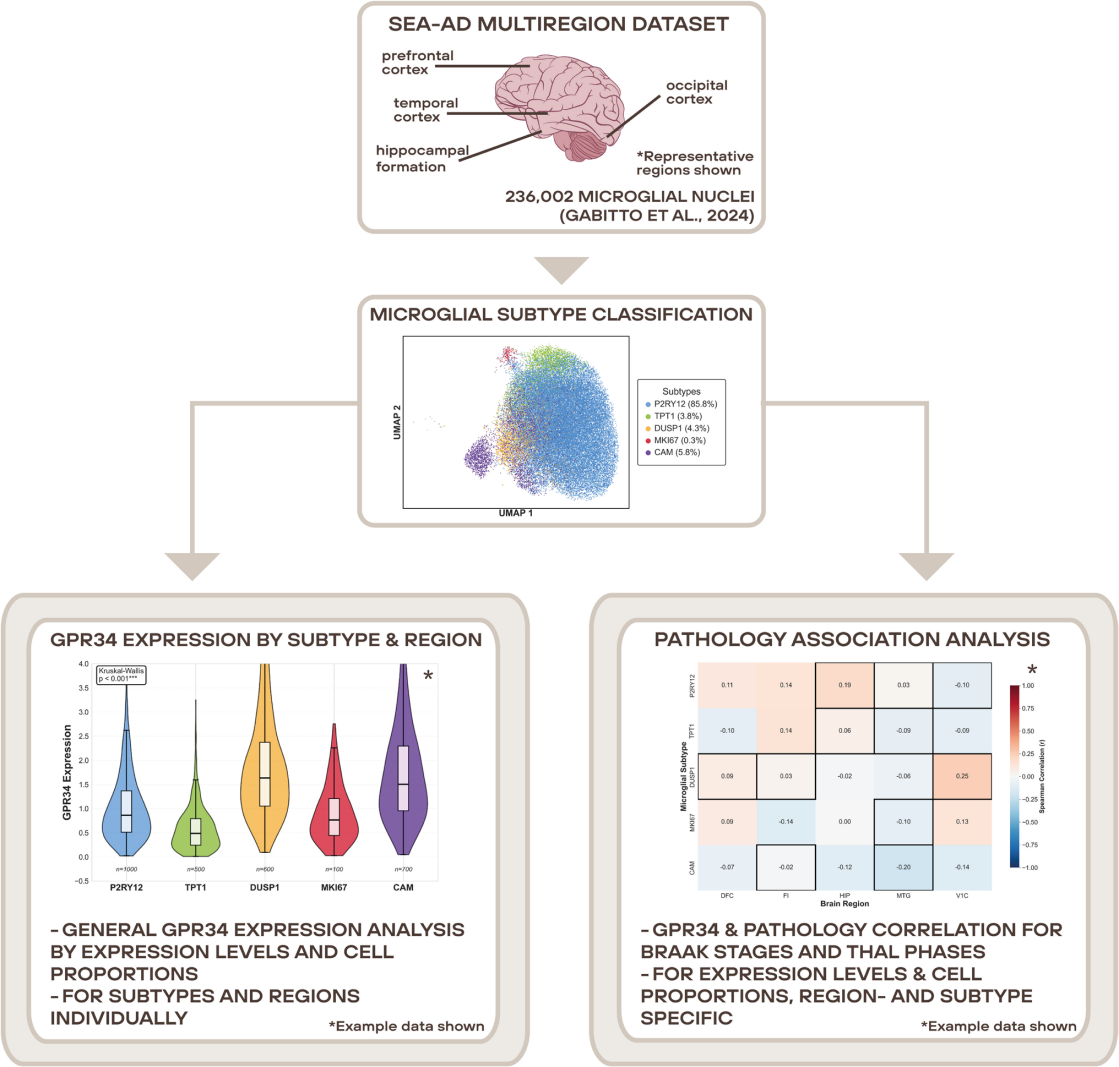
Computational workflow for single-nucleus RNA sequencing analysis of microglial GPR34 expression in AD. Analytical pipeline for single-nucleus RNA sequencing (snRNA-seq) data. Preprocessed data from 236,002 microglial nuclei across 10 brain regions were analyzed using Scanpy. Nuclei were classified into five microglial subtypes based on marker gene expression, and GPR34 expression was extracted and stratified by neuropathological staging (Braak stages/Thal phases), brain region, and subtype. Statistical analyses included non-parametric group comparisons (Kruskal‒Wallis, Mann‒Whitney U), proportion testing (Chi-square), and correlation analyses (Spearman), with FDR correction. Results were visualized to reveal regional, subtype-specific, and pathology-associated patterns of GPR34 expression. This figure was created using Adobe Photoshop v26.0

Analysis was performed in Python (v3.9) using Scanpy (v1.10.1) and associated packages. Microglial subtypes were classified using marker genes from Mathys et al. [79]: P2RY12, TPT1 (homeostatic), DUSP1 (activated), MKI67 (proliferating), and CNS-associated macrophages (CAMs). Complete marker gene lists are provided in Table 2. For each nucleus, a subtype-specific expression score was calculated as the mean log-normalized expression across all marker genes within the respective gene set. Each nucleus was subsequently assigned to the subtype with the highest expression score. GPR34 expression was extracted for each nucleus and stratified by donor, brain region, microglial subtype, Braak stage and Thal phase. Workflow for snRNA-seq analysis is illustrated in Fig. 3.

**Table 2:** Marker genes for microglial subpopulation classification

Microglial subpopulation	Description	Marker genes
MKI67	Proliferating microglia	NSD2, RFC3, SMC4, ATAD2, EZH2, HELL, CIT, NCAPG2, BRIP1, DIAPH3
P2RY12	Homeostatic microglia	ITPR2, APBB1IP, FRMD4A, NAV3, RASSF8, FAM149A, MCF2L, SYNDIG1, P2RY12, KBTBD12
DUSP1	Activated microglia	SRGN, PFKFB3, HIF1A, RGS1, DUSP1, FOS, GPR183, SLC2A3, SLC7A5, CD83
TPT1	Resting/homeostatic microglia	RPS8, RPL11, RPS24, RPS11, RPL19, RPS6, PLEKHA7, RPL32, RPS20, RPL23A
CAMs	CNS-associated macrophages	MYO5A, STARD12, RGL1, ITSN1, MS4A4A, CD163, MCTP1, F13A1, EY2A, SIGLEC1

Marker gene sets used to define functional microglial subpopulations. Gene lists were derived from Mathys et al. (2024), Supplementary Fig. 24f [79]. CNS = central nervous system

### Statistical analysis

Statistical analyses were performed using Mathematica and IBM® SPSS® Statistics (v29; IBM Corp.). Data were visualized using box plots, scatter plots, and line plots. Descriptive statistics were calculated, and data were tested for normal distribution using the Shapiro‒Wilk test. Non-parametric tests were applied throughout due to non-normal distributions. Group differences were evaluated using Mann‒Whitney U (unpaired), Wilcoxon signed-rank (paired), and Kruskal‒Wallis with Dunn’s post hoc (multiple unpaired) tests. Associations were assessed with Spearman’s rank correlation coefficient (r). Effect sizes were interpreted based on the absolute value of *r* (|*r*|) as follows: very strong (≥ 0.80), strong (0.60‒0.79), moderate (0.40‒0.59), weak (0.20‒0.39), and very weak (< 0.20) [4]. The independent contributions of donor age, PMI, and sex to GPR34 signal counts were examined using multivariable linear regression; model fit was assessed by adjusted *R*^2^.

For snRNA-seq data, all inferential tests for group differences and associations with AD pathology were performed at the donor level. Donor-level mean GPR34 expression was restricted to GPR34-expressing (GPR34+) cells and calculated only for groups comprising at least five GPR34+ cells. Subtype differences in the proportion of GPR34+ microglia and in mean GPR34 expression were assessed across four microglial subtypes with sufficient donor coverage (P2RY12, TPT1, DUSP1, CAM) using the Friedman test (i.e., paired across subtypes within donors), with pairwise follow-up using Wilcoxon signed-rank tests and Benjamini‒Hochberg false discovery rate (FDR) correction. MKI67 microglia were analyzed descriptively due to sparse donor coverage. Regional differences were assessed at the donor level using the Kruskal‒Wallis test across all 10 regions and the Friedman test restricted to the three regions with the greatest donor overlap (DFC, MEC, MTG; *n* = 77 donors). Associations with Braak stage and Thal phase were evaluated using two-sided Spearman rank correlations on donor-level summaries (proportion of GPR34+ cells and mean log-expression in GPR34+ cells), with Benjamini‒Hochberg FDR correction applied across all region × subtype tests within each staging framework. Region × subtype combinations with fewer than eight donors were excluded. To assess potential confounding by demographic variables, donor-level GPR34 measures were tested for associations with age using Spearman correlation and sex using Mann‒Whitney U tests and ordinary least squares regression models, analyzed separately for Braak stage and Thal phase. Statistical significance was defined as p < 0.05 (FDR-corrected p < 0.05 for multiple testing).

## Results

### Microglial phenotype and GPR34 expression in relation to AD-related deposits

To investigate how AD-related deposits (Aβ and p-tau) affect microglial phenotype and GPR34 expression, we performed combined RNA in situ hybridization and double immunofluorescence labeling in post-mortem brain tissue from 20 human donors (Table 1). We used a human GPR34 RNA probe in combination with Iba1/Aβ or Iba1/AT8 antibody cocktails. In the ROI’s (EC and TC), all Iba1-positive microglia were identified, and their density, morphology, and GPR34 expression were quantified in relation to local Aβ- and AT8-positive deposits. Following the combined FISH-IF procedure, one donor (case #4) was excluded due to unreliable Iba1 immunolabeling, which impeded visualization of microglia. This donor had died of fulminant hepatic failure, a condition known to interfere with microglial detection in post-mortem brain tissue [69]. In the remaining 19 cases, 187,670 microglia were analyzed, comprising 116,138 cells in the EC and 71,532 in the TC. All automatic detections were manually reviewed and corrected where necessary.

### Case classification by pathological burden

Each case was evaluated separately for Aβ and p-tau burdens, defined as the percentage of tissue area occupied by Aβ- or AT8-positive deposits. Cases were then classified into three Aβ categories (low, moderate, high) and four p-tau categories (low, moderate, high, severe). Median values of Aβ and p-tau burdens per category, as well as descriptive statistics for all quantified parameters, are summarized in Table 3.

**Table 3:** Donor characteristics and microglial parameters in categories with varying Aβ and p-tau burden

Aβ burden		Low	Moderate	High	P-tau burden		Low	Moderate	High	Severe
Age (y)		71.0 (20.0)	81.5 (6.0)	86.0 (6.0)	Age (y)		72.0 (15.5)	71.0 (2.0)	81.0 (6.0)	86.0 (4.0)
PMI (h)		48.0 (31.0)	48.0 (10.0)	18.5 (16.5)	PMI (h)		48.0 (19.0)	72.0 (20.0)	24.0 (15.0)	18.5 (8.5)
Aβ burden (%)	EC	0.0002 (0.0018)	0.2297 (0.6182)	4.3688 (2.4723)	P-tau burden (%)	EC	0.1599 (0.0978)	0.4587 (0.8090)	1.0692 (1.2798)	3.5068 (2.2678)
	TC	0.0000 (0.0005)	0.7817 (0.2567)	5.5756 (2.6165)		TC	0.0613 (0.0332)	0.0512 (0.2023)	1.1101 (0.3466)	2.4515 (1.4746)
MG density (n/mm^2^)	EC	69.62 (33.78)	80.48 (11.98)	71.38 (39.99)	MG density (n/mm^2^)	EC	79.97 (27.06)	65.12 (44.09)	71.13 (30.31)	84.01 (68.85)
	TC	54.76 (21.36)	96.43 (23.84)	74.82 (34.23)		TC	77.68 (29.68)	58.32 (37.92)	77.57 (29.68)	84.45 (84.51)
GPR34 signal count (*n*)	EC	12.50 (10.76)	19.78 (8.02)	31.12 (17.63)	GPR34 signal count (*n*)	EC	19.54 (3.40)	13.63 (4.59)	21.07 (2.92)	28.11 (30.74)
	TC	14.52 (7.95)	16.60 (10.11)	34.78 (13.89)		TC	15.28 (0.82)	13.43 (3.56)	24.89 (12.05)	30.89 (20.83)
MG soma size (µm^2^)	EC	107.65 (15.24)	106.85 (37.55)	103.90 (23.80)	MG soma size (µm^2^)	EC	93.51 (17.56)	88.97 (12.59)	97.99 (6.34)	95.99 (15.00)
	TC	100.76 (6.44)	92.88 (26.26)	100.10 (22.31)		TC	78.96 (12.24)	93.27 (6.46)	92.58 (10.18)	93.37 (22.15)
MG process length (µm)	EC	12.07 (5.10)	11.05 (7.73)	12.07 (2.91)	MG process length (µm)	EC	8.95 (3.73)	12.40 (4.51)	7.16 (3.65)	11.33 (1.72)
	TC	10.49 (5.44)	10.66 (4.42)	12.26 (4.81)		TC	13.47 (4.65)	10.89 (2.42)	6.80 (1.93)	10.16 (6.16)
Aβ deposit distance (µm)	EC	4,942.37 (3,129.92)	934.98 (1,215.94)	66.37 (50.35)	P-tau deposit distance (µm)	EC	53.69 (96.18)	27.40 (19.78)	13.0 (5.53)	5.87 (2.55)
	TC	10,545.27 (12,500.77)	436.83 (508.87)	53.48 (12.90)		TC	143.17 (80.08)	92.59 (76.21)	14.7 (5.35)	8.00 (2.65)
MG count (*n*)	EC	18,859	13,604	27,369	MG count (*n*)	EC	4813	18,612	10,598	22,283
	TC	12,404	7991	17,301		TC	5367	8880	6946	12,643
Cases (*n*)		7	4	8	Cases (*n*)		3	5	5	6

Median values with interquartile ranges (in parentheses) are given. EC = entorhinal cortex, TC = temporal cortex, Age (y) = age at death in years (y), PMI (h) = post-mortem interval in hours (h), Aβ burden (%) = percentage of tissue area occupied by amyloid-β, P-tau burden (%) = percentage of tissue area occupied by hyperphosphorylated tau, MG density (n/mm^2^) = regional density of microglia in cells per mm^2^, GPR34 signal count (*n*) = number of GPR34 mRNA signals per cell, MG soma size (µm^2^) = microglial soma area in µm^2^, MG process length (µm) = microglial proximal process length in µm, Aβ deposit distance (µm) = median distance of each microglia to the nearest Aβ deposit, P-tau deposit distance (µm) = median distance of each microglia to the nearest p-tau deposit, MG count (*n*) = total number of microglia, Cases (*n*) = number of cases per category

### Microglial density and morphology remain largely unchanged

**Fig. 4:**
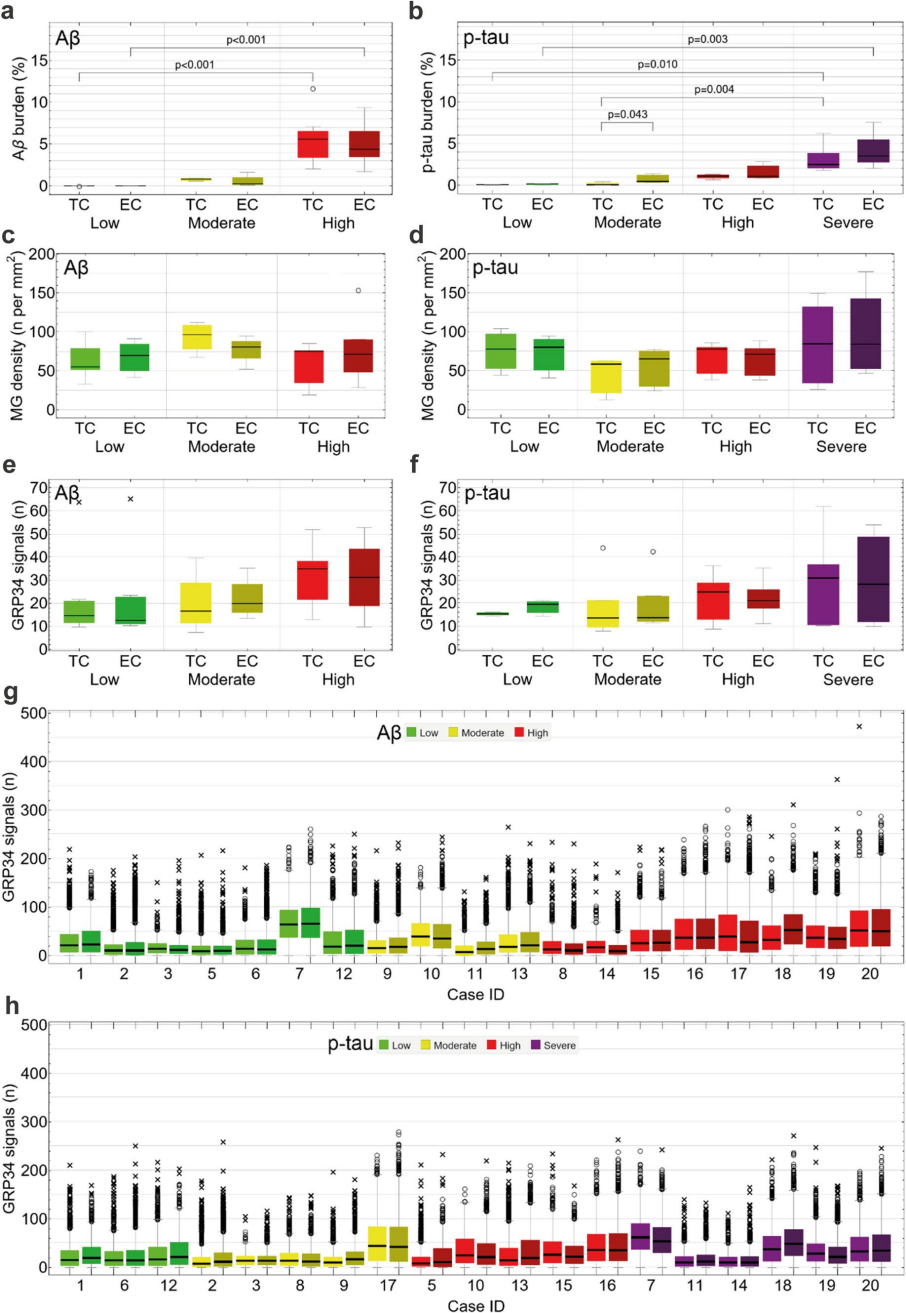
Regional deposit burden, microglial density, and GPR34 mRNA signal counts across burden categories and individual cases. **a** and **b** Extent of Aβ (a) and p-tau (b) deposition expressed as percentage area in the entorhinal cortex (EC) and temporal cortex (TC), grouped according to the three (Aβ) and four (p-tau) burden categories, respectively. **c** and **d** Regional microglial (MG) density in TC and EC across Aβ (**c**) and p-tau (d) burden categories. Densities are expressed as cells per mm^2^. MG density showed no consistent association with Aβ or p-tau burdens across categories. **e** and **f** GPR34 signal counts per Iba1-positive microglial cell in TC and EC across Aβ (e) and p-tau (f) burden categories. Each data point reflects the median value per case, calculated across all microglia within the respective region. While GPR34 signal counts showed a tendency to increase with higher Aβ and p-tau burdens, these trends did not reach statistical significance. **g** and **h** Single-cell-level analysis of Iba1-labeled microglia reveals pronounced inter- and intra-individual heterogeneity in GPR34 signal counts across all burden categories. Box plots show the distribution of GPR34 signal counts per cell for each case in TC (left) and EC (right), grouped by Aβ (g) and p-tau (h) burden categories. Boxes indicate the interquartile range (IQR) with median; whiskers extend to 1.5× IQR; circles mark outliers and crosses denote extreme values. Statistically significant group differences are indicated by brackets with corresponding p values. Plots were generated using Wolfram Mathematica v14.0

Regional microglial density (cells/mm^2^) was calculated on 10 µm sections by dividing the number of Iba1-positive microglia by the respective ROI area in EC and TC. Median microglial density did not differ significantly across Aβ or p-tau categories, either within the EC or TC, or between the two regions within any given category (Fig. 4). No evidence of spatial clustering was observed using Iba1 identification. Inter-case variability in microglial density was comparable across all categories.

An inverse correlation between soma size and proximal process length was consistently observed across all categories and cases, with larger somata associated with shorter processes (Aβ: *r* = −0.61, *p* = 0.006; p-tau: *r* = −0.35, *p* = 0.139), consistent with established microglial phenotypes ranging from ramified-resting to amoeboid-activated states [115]. Morphometric analyses focused on proximal branches from 2D Extended Depth of Field (EDOF) projections and processes assigned within 75 px (~12.9 µm) of the soma; more distal branching was not captured. Region-level soma size and proximal process length remained unchanged across categories: neither soma size nor proximal process length differed significantly across Aβ or p-tau categories.

GPR34 signal counts did not correlate with soma size or proximal process length in either staining set or brain region (not shown). A significant positive correlation between regional microglial density and GPR34 signal counts was observed in the EC for Aβ-stained tissue (*r* = 0.64, *p* = 0.003), but not in the TC or AT8-stained sections (not shown).

### Single-cell GPR34 expression is highly variable

GPR34 expression did not show a robust association with Aβ or p-tau burdens. Although median GPR34 signal counts tended to increase from low to high Aβ burden in both EC (12.50 [10.76], 19.78 [8.02], 31.12 [17.63]) and TC (14.52 [7.95], 16.60 [10.11], 34.78 [13.89]), this trend was inconsistent across individual cases and did not reach statistical significance (Fig. 4e). For example, a low-burden case (#7) exhibited exceptionally high GPR34 counts in both regions (Fig. 4g). A similar, non-significant trend was observed across p-tau categories. Median GPR34 signal counts showed a slight decrease from low to moderate p-tau burden, followed by a gradual increase in high and severe categories. EC median values were 19.54 [3.40], 13.63 [4.59], 21.07 [2.92], and 28.11 [30.74] for low, moderate, high, and severe p-tau burdens, respectively; TC median values were 15.28 [0.82], 13.43 [3.56], 24.89 [12.05], and 30.89 [20.83]. In the moderate p-tau category—where tau pathology was largely confined to the EC—GPR34 signal counts did not differ between EC and TC.

**Fig. 5:**
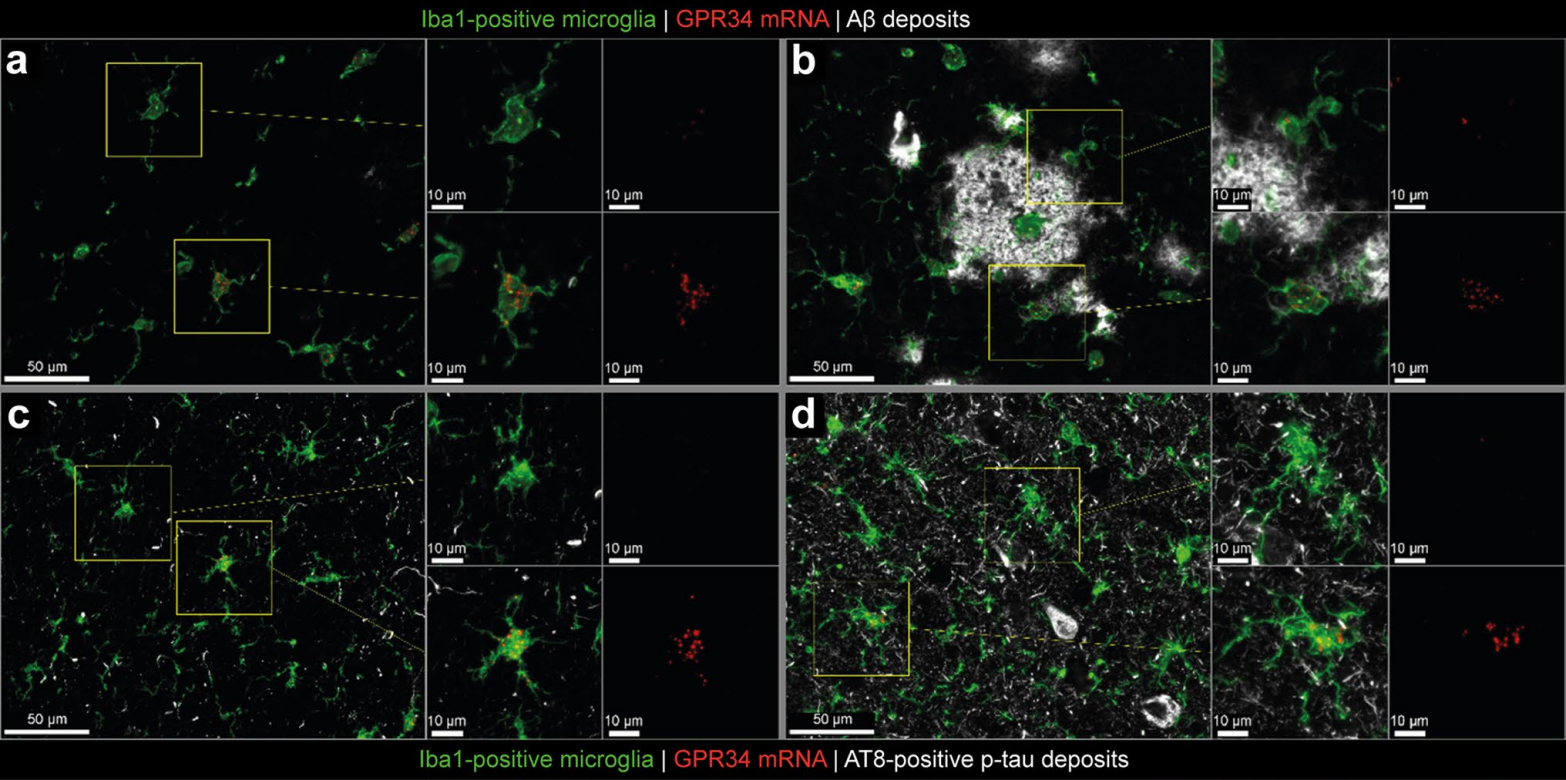
The number of GPR34 signals in individual microglia is independent of local Aβ or p-tau deposits. **a** and **b** High-resolution images from whole-slide scans of medial temporal lobe cortex tissue, stained by combined fluorescent in situ hybridization (FISH) and immunofluorescence double labeling using Iba1 antibody for microglia (Alexa Fluor 647; green), Aβ antibody for Aβ deposits (Alexa Fluor 488; white), and a GPR34 mRNA probe (FISH, Cy3; red). GPR34 signal counts varied markedly among microglia in regions without detectable Aβ deposits (a) as well as in regions with abundant Aβ deposition (b). **c** and **d** Corresponding staining for AT8-positive deposits (AT8, Alexa Fluor 488; white) revealed a similar variability in GPR34 signal counts among Iba1-positive microglia in regions with low (c) and high (d) p-tau burdens. **a**‒**d** Each panel provides an overview (left) and enlarged sections (right), with representative microglia displayed in individual channels (Iba1 on the left, GPR34 on the right). GPR34 signal counts differ substantially between neighboring microglia, irrespective of the presence or absence of adjacent Aβ or p-tau deposits. This figure was created using Adobe Photoshop v26.0

**Fig. 6:**
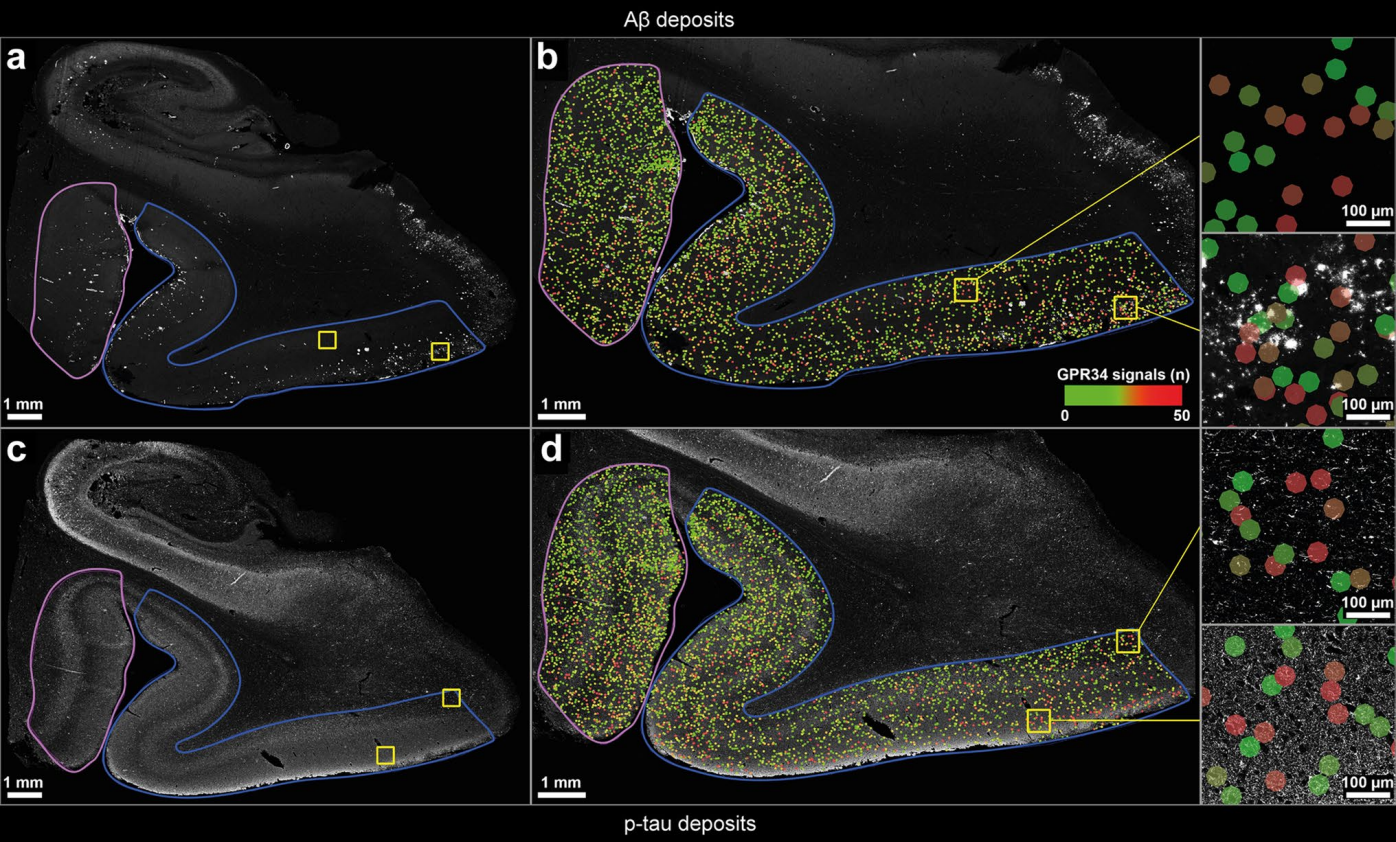
Spatial mapping of microglial GPR34 signal counts in relation to Aβ and p-tau deposits. **a** and **c** Whole-slide scans of adjacent sections from the medial temporal lobe cortex of case #16 (with moderate Aβ burden and severe p-tau burden) illustrate regional differences in the distribution of Aβ- and AT8-positive deposits. Immune reactivity for Aβ (a) and AT8 (c) is labeled with Alexa Fluor 488 dye and shown in white. The entorhinal cortex (EC) and temporal cortex (TC) are delineated in blue and pink, respectively. **b** and **d** Corresponding sections from (a) and (c), overlaid with single-cell scatter plots showing the spatial positions of Iba1-labeled microglia within the EC and TC. Each dot represents an individual microglial cell, colored according to the number of GPR34 mRNA signals detected per cell (green = low, red = high; scale 0–50). Yellow squares mark two regions within the EC, enlarged on the right. Upper enlargements depict an area with low Aβ (b) or p-tau (d) burdens, respectively; lower enlargements show areas with high Aβ (b) or p-tau (d) burdens, respectively. No evident spatial association was observed between microglial density or GPR34 signal counts and the distribution of Aβ- or p-tau deposits. Instead, GPR34 signal counts displayed pronounced cell-to-cell variability that appeared largely independent of local deposit burden. This figure was created using Adobe Photoshop v26.0

Inter-individual variability increased with pathology burden, and intra-individual heterogeneity was substantial: within the same region, microglia displayed wide ranges of GPR34 signal counts (Fig. 4g, h). Cells with low and high GPR34 counts were frequently found in close spatial proximity, independent of nearby Aβ- or p-tau deposits (Fig. 5). Spatial mapping confirmed a random distribution of GPR34 expression throughout the EC and TC (Fig. 6).

### Pathology burden influences microglia-to-deposit distance

Increasing Aβ or p-tau burdens were associated with reduced distances between microglia and the nearest deposit (Table 3). In high Aβ cases, microglia were significantly closer to deposits than in low-burden cases (EC: *p* < 0.001; TC: *p* < 0.001). Similarly, in severe p-tau cases, microglia were closer to p-tau deposits than in low (EC: *p* = 0.004; TC: *p* = 0.006) or moderate (EC: *p* = 0.018; TC: *p* = 0.005) categories. Within moderate p-tau cases, microglia in the EC were closer to deposits than in the TC (*p* = 0.043), reflecting the regional progression of tau pathology in AD [20].

Higher pathology burden corresponded to a greater proportion of microglia located near deposits, while lower-burden categories showed broader distributions. Notably, reduced median distances were accompanied by narrower interquartile ranges, indicating more uniform microglial positioning around deposits at advanced stages.

### Distance-dependent microglial responses

**Fig. 7:**
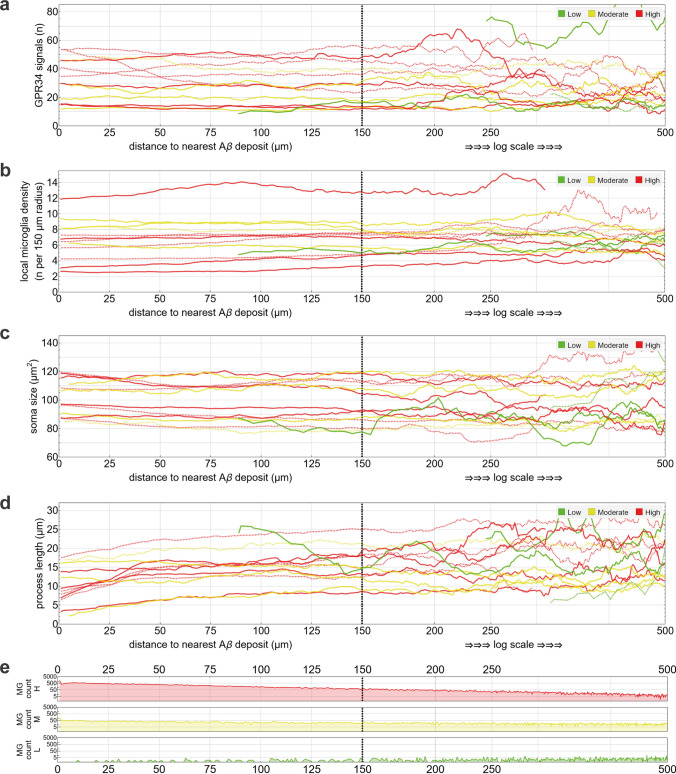
Distance-resolved analysis of microglial properties in relation to Aβ deposits. **a**‒**d** GPR34 mRNA signal counts (**a**), local microglial density (**b**), microglial soma size (**c**), and proximal process length (**d**) plotted as a function of distance to the nearest Aβ deposit. For each microglial cell, the distance to the nearest deposit was calculated and values were aggregated in 1-µm-distance bins. Lines represent individual cases and show the case-level median within each bin, calculated across all microglia located at the corresponding distance from the nearest deposit. Dotted lines indicate female donors, solid lines indicate male donors. Cases are color-coded according to Aβ burden category (low, moderate, high). Profiles were smoothed using a mean filter, and truncated at both ends to reduce edge effects, which were most pronounced in low-burden cases with sparse sampling at short distances. The x-axis is plotted on a linear scale between 0 and 150 µm, capturing the region most relevant for potential deposit-associated microglial effects, and on a logarithmic scale between 150 and 500 µm to visualize more distal spatial patterns. The vertical dashed line indicates the transition between linear and logarithmic scaling. Microglial GPR34 counts, local microglial density, and soma size showed substantial inter-case variability without consistent distance-related changes, whereas proximal process length was reduced at shorter distances to Aβ deposits. **e** Number of Iba1-positive microglia contributing to the distance profiles, shown as cell counts per 1-µm bin for each Aβ burden category. The x-axis follows the same combined linear/logarithmic scaling as in panels **a**‒**d**. In low-burden cases, only few microglia are present at short distances (0‒150 µm), resulting in sparse green curves in this range, whereas in high-burden cases, the higher Aβ burden is associated with more microglia near deposits. Plots were generated using Wolfram Mathematica v14.0

**Fig. 8:**
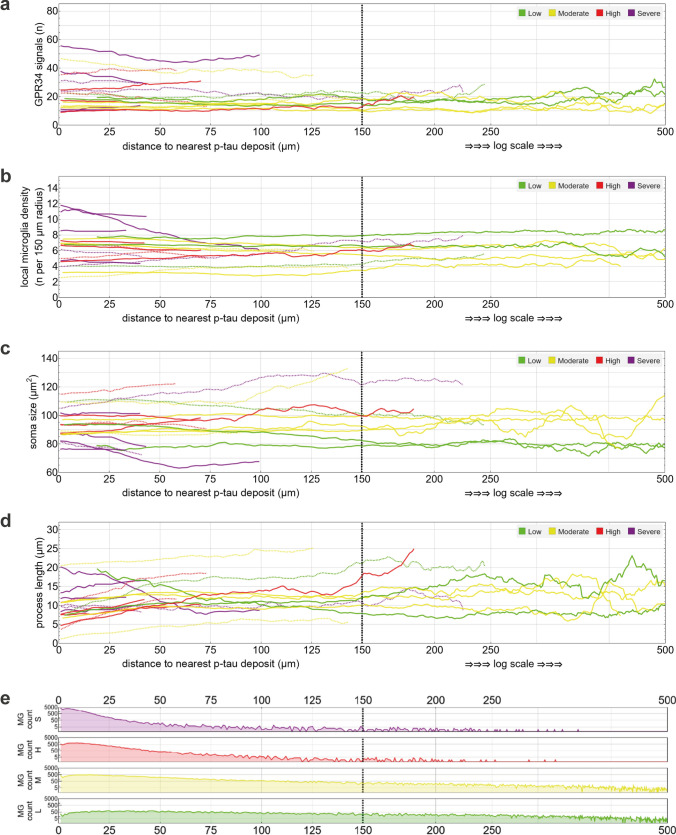
Distance-resolved analysis of microglial properties in relation to p-tau deposits. **a**‒**d** GPR34 mRNA signal counts (**a**), local microglia density (**b**), microglial soma size (**c**), and proximal process length (**d**) plotted as a function of distance to the nearest p-tau deposit. For each microglial cell, the distance to the nearest deposit was calculated and values were aggregated in 1-µm-distance bins. Lines represent individual cases and show the case-level median within each bin, calculated across all microglia located at the corresponding distance from the nearest deposit. Dotted lines indicate female donors, solid lines indicate male donors. Cases are color-coded according to p-tau burden category (low, moderate, high, severe). Profiles were smoothed using a mean filter, and truncated at both ends to reduce edge effects. The x-axis is plotted on a linear scale between 0 and 150 µm, capturing the region most relevant for potential deposit-associated microglial effects, and on a logarithmic scale between 150 and 500 µm to visualize more distal spatial patterns. The vertical dashed line indicates the transition between linear and logarithmic scaling. Microglial GPR34 counts, local microglial density, soma size, and proximal process length showed substantial inter-case variability without a consistent distance-dependent relationship to p-tau deposit proximity. **e** Number of Iba1-positive microglia contributing to the distance profiles, shown as cell counts per 1-µm bin for each p-tau burden category. The x-axis follows the same combined linear/logarithmic scaling as in panels **a**‒**d**. Plots were generated using Wolfram Mathematica v14.0

Microglia were binned by distance to the nearest Aβ or p-tau deposit to correlate microglial properties (GPR34 signal counts, local density, soma size, and proximal process length) with deposit proximity (Figs. 7, 8).

Distance-resolved single-cell analysis (0–150 µm from the nearest deposit [19, 49, 53, 111]) revealed four results: (i) GPR34 signal counts showed no consistent distance-dependent changes for either Aβ or p-tau deposits, though substantial inter-case variability was observed (Figs. 7a, 8a). (ii) Local microglia density remained stable across all distances, showing no distance-dependent changes or clustering around Aβ or p-tau deposits within a radius of 0–150 µm from the nearest deposit, with only minor exceptions in severe-burden cases (Figs. 7b, 8b). The greatest variability between cases was observed in categories with the highest deposit burden, while the remaining categories showed comparatively uniform microglia densities. (iii) Microglial soma size remained largely stable in relation to Aβ deposits (Fig. 7c), whereas proximal process length increased with increasing distance, particularly within 0–100 µm (Fig. 7d). Median proximal process length increased from 10.4 µm at 1 µm to 15.9 µm at 100 µm, with the sharpest increase occurring between 0 and 50 µm. Only minor exceptions were observed in low-burden cases with few microglia. In the p-tau cohort, both soma size and proximal process length exhibited distance-related shifts, but the direction and magnitude of changes were highly variable between cases (Fig. 8c, d). Some cases displayed increased proximal process length near deposits, others decreased, and some remained unchanged. No consistent relationship with deposit proximity or burden category was detected. (iv) Consistent with the distribution of pathological deposits, the absolute number of microglia located near deposits was higher in cases with greater pathological deposition than in cases with lower deposition (Figs. 7e, 8e).

### GPR34 expression in relation to donor age, PMI, and sex

**Fig. 9:**
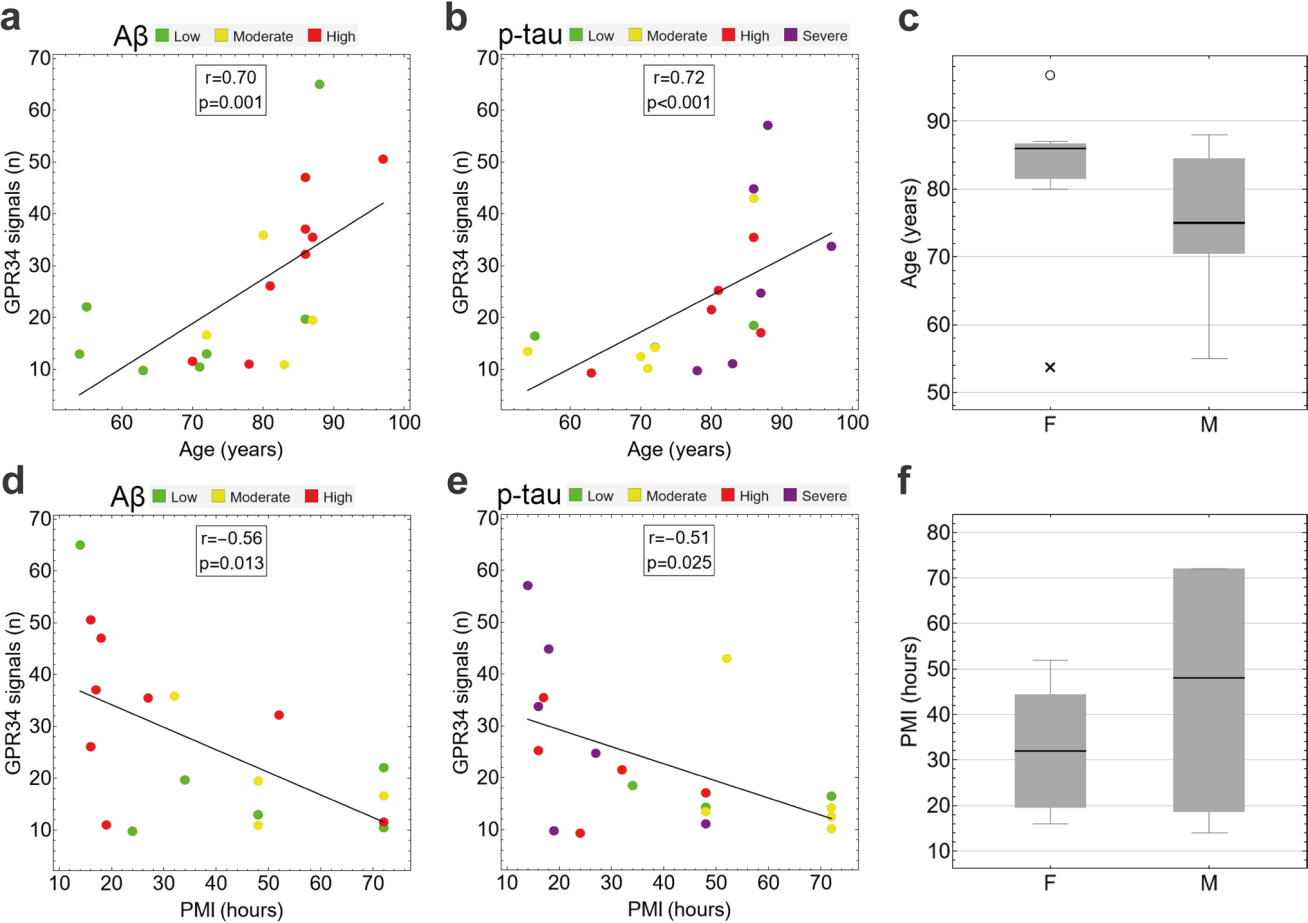
Donor age and post-mortem interval correlate with GPR34 signal counts and differ between sexes. **a** and **b** GPR34 signal counts per microglial cell plotted against donor age in Aβ- (a) and p-tau-stained (b) sections. **c** Donor age by sex. **d** and **e** GPR34 signal counts plotted against post-mortem interval (PMI) before brain processing in Aβ- (c) and p-tau-stained (d) sections. **f** PMI by sex. In the scatter plots (a, b, d, e), each data point represents the median GPR34 signal count per case, calculated across all Iba1-labeled microglia within the entorhinal cortex and temporal cortex. Colors indicate Aβ burden categories (green: low; yellow: moderate; red: high), or p-tau burden categories (green: low; yellow: moderate; red: high; purple: severe). Solid lines indicate linear regression fits; Spearman’s rank correlation coefficients (*r*) and corresponding p values are shown. In box plots (c, f), boxes indicate the interquartile range with the median line; whiskers extend to 1.5× IQR; circles denote outliers and crosses extreme values. GPR34 signal counts were positively associated with donor age and negatively associated with PMI. Female donors were on average older and had shorter PMI than male donors. Plots were generated using Wolfram Mathematica v14.0

In our FISH-based analysis, GPR34 signal counts were positively correlated with donor age (EC + TC: Aβ: *r* = 0.70, *p* = 0.001; p-tau: *r* = 0.72, *p* < 0.001; Fig. 9a, b), and negatively correlated with PMI (Aβ: *r* = −0.56, *p* = 0.013; p-tau: *r* = −0.51, *p* = 0.025; Fig. 9d, e), with consistent correlations observed within EC and TC (not shown). Female donors showed consistently higher GPR34 signal counts than male donors across Aβ and p-tau burden categories in both regions, except in the EC among cases with severe p-tau burden; none of these differences reached statistical significance (Supplementary Fig. S1, Supplementary Table S4). Female donors were on average older (median 86 [5.25] vs. 75 [14.0] years) and had a shorter PMI (median 32 [25] vs. 48 [53.5] h) than male donors (Fig. 9c, f).

In the SEA-AD snRNA-seq dataset, donor age was likewise positively correlated with both the proportion of GPR34+ microglia (*r* = 0.25, *p* = 0.020) and mean GPR34 expression (*r* = 0.25, *p* = 0.023; Supplementary Fig. S2). PMI was negatively correlated with GPR34+ proportions (*r* = −0.23, *p* = 0.032) but not with mean expression (*r* = −0.12, p = 0.291; Supplementary Fig. S3). Consistent with the FISH-based findings, female donors showed a non-significant trend toward higher proportions of GPR34+ microglia than male donors (median 48.3% vs 45.9%; mean 48.7% vs 43.0%; Mann‒Whitney U = 1032, *p* = 0.082; Supplementary Fig. S4a), with no significant sex differences detected within any microglial subtype after multiple-testing correction (proportion: minimum FDR-adjusted *q* = 0.17; expression: minimum FDR-adjusted *q* = 0.50, Supplementary Fig. S4b, f).

In multivariable analyses including donor age, PMI, and sex, none of the three variables remained independently significant in either cohort (FISH: all *p* ≥ 0.09; SEA-AD: all *p* ≥ 0.56).

### GPR34 expression across microglial subtypes and brain regions

**Fig. 10:**
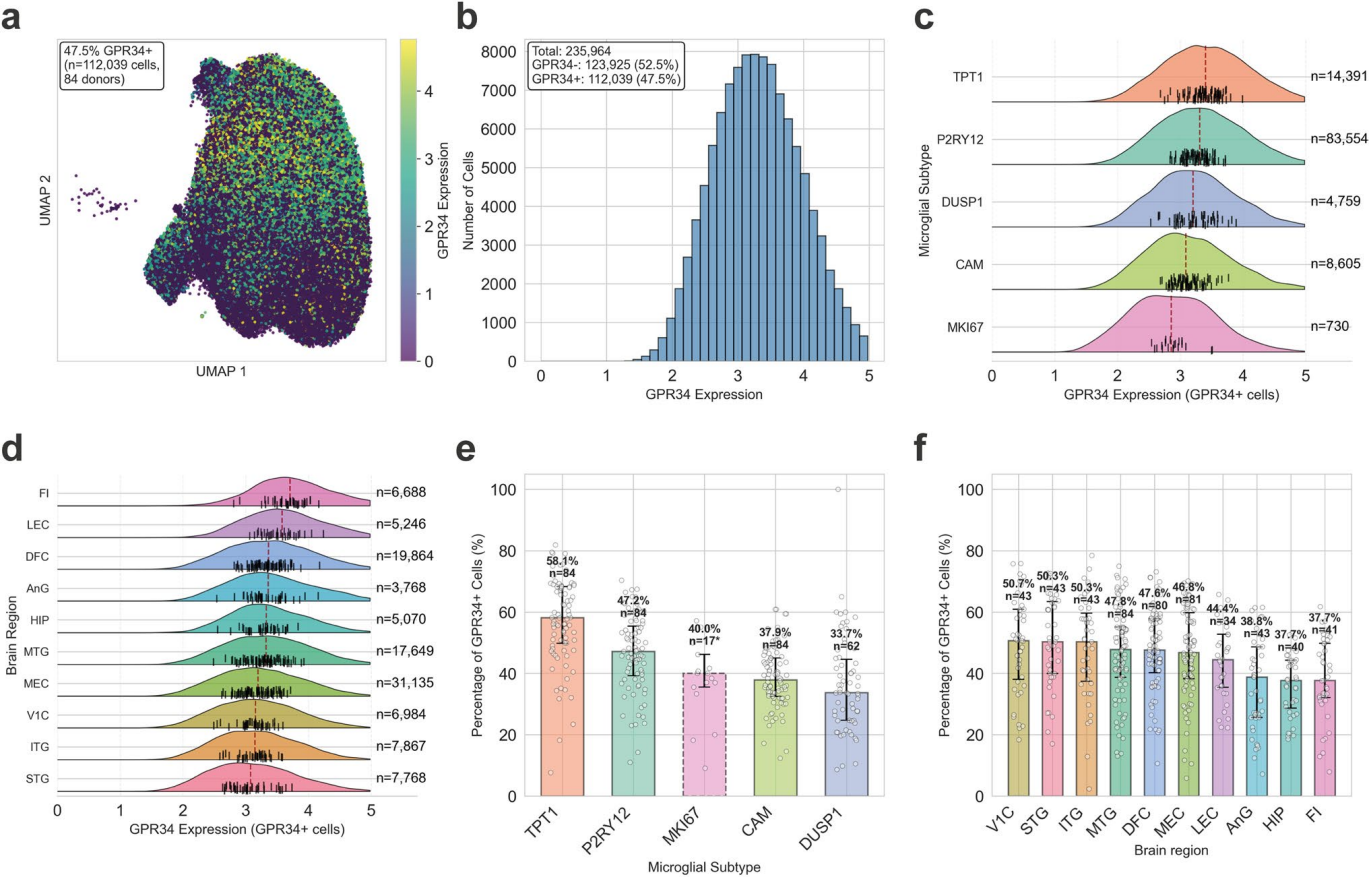
GPR34 expression across microglial subtypes and brain regions. **a** UMAP projection of microglial cells colored by GPR34 expression intensity, demonstrating marked expression heterogeneity across the population. Overall, 47.5% of microglia were GPR34-positive (GPR34+; expression > 0), corresponding to 112,048 of 236,002 nuclei. **b** Distribution of GPR34 expression levels among GPR34+ microglia. **c** Ridge plots showing the distribution of GPR34 expression across microglial subtypes (GPR34+ cells only), ordered by median expression. Red dashed lines indicate median values. TPT1 microglia (resting/homeostatic) exhibited the highest median expression, while MKI67-positive (proliferating) microglia showed the lowest. At the donor level, mean GPR34 expression differed significantly across P2RY12, TPT1, DUSP1, and CAM microglia (Friedman χ^2^ = 55.31, *p* < 0.001). **d** Ridge plots showing the distribution of GPR34 expression across brain regions (GPR34+ cells only). Median expression varied across regions, with the frontoinsular cortex (FI) showing the highest donor-level median (3.74) and the superior temporal gyrus (STG) the lowest (3.05). Regional differences were significant at the donor level when all 10 regions were compared (Kruskal‒Wallis *H* = 142.25, *p* < 0.001); a paired comparison restricted to the three regions with the most complete donor overlap (DFC, MEC, MTG; *n* = 77 donors) also revealed significant regional variation (Friedman χ^2^ = 21.89, *p* < 0.001). **e** Donor-level percentage of GPR34+ microglia by subtype. TPT1 microglia showed the highest frequency (58.1%), while DUSP1 microglia showed the lowest (33.7%). Friedman test indicates significant heterogeneity in GPR34+ cell proportions across subtypes (Friedman χ^2^ = 89.36, *p* < 0.001). **f** Donor-level percentage of GPR34+ cells by brain region, demonstrating regional variation ranging from 37.7% (HIP, FI) to 50.7% (V1C). Regional differences in GPR34+ frequencies were significant when comparing all 10 regions (Kruskal‒Wallis *H* = 53.50, *p* < 0.001) but not detectable within the same donor across DFC, MEC, and MTG (Friedman χ^2^ = 1.25, *p* = 0.536). All inferential statistics were computed at the donor level (one observation per donor per subtype or region). Cell-level layers in panels a–d are shown for descriptive context and are overlaid by donor layer. Density curves in c‒d were estimated using Gaussian kernel density estimation with Scott’s bandwidth selection method. Plots were created using Python v3.9. *Abbreviations: MTG – middle temporal gyrus, STG – superior temporal gyrus, V1C – primary visual cortex, MEC – medial entorhinal cortex, LEC – lateral entorhinal cortex, HIP – hippocampal formation, ITG – inferior temporal gyrus, AnG – angular gyrus, FI – frontoinsular cortex, DFC – dorsolateral frontal cortex*

Analysis of 236,002 microglia revealed marked heterogeneity in GPR34 expression (Fig. 10). Overall, 47.5% of microglia were GPR34+, but frequency varied significantly by subtype (Friedman χ^2^ = 89.36, *p* < 0.001; *n* = 62 donors with complete data across P2RY12, TPT1, DUSP1, and CAM): homeostatic TPT1 and P2RY12 microglia showed the highest per-donor proportion (median 58.1%, IQR 49.8‒68.3% and 47.2%, IQR 39.2‒55.4%, respectively), activated DUSP1 microglia and CNS-associated macrophages the lowest (33.7%, IQR 24.7‒44.5% and 37.9%, IQR 32.4‒45.0%), with proliferating MKI67 microglia (40.0%, IQR 35.5‒46.2%) showing intermediate prevalence (Fig. 10e).

GPR34+ proportions also differed significantly across brain regions (Kruskal‒Wallis *H* = 53.50, *p* < 0.001), with hippocampal formation and FI showing the lowest frequency of GPR34+ microglia (both 37.7%) and V1C the highest (50.7%). Other regions displayed intermediate proportions, including STG (50.3%), ITG (50.3%), LEC (44.4%) and DFC (47.6%; Fig. 10f). When restricted to the three regions with the most complete donor overlap (DFC, MEC, MTG; *n* = 77 donors), no within-donor regional difference in GPR34+ proportion was detectable (Friedman χ^2^ = 1.25, *p* = 0.54), while expression intensity among GPR34+ cells showed significant within-donor regional variation (Friedman χ^2^ = 21.89, *p* < 0.001). Expression intensity also differed significantly by subtype (Friedman χ^2^ = 55.31, *p* < 0.001) and across all 10 regions (Kruskal‒Wallis *H* = 142.25, *p* < 0.001), with subtype ordering broadly matching that observed for GPR34+ proportions (Fig. 10c, d).

### GPR34 expression during disease progression

**Fig. 11:**
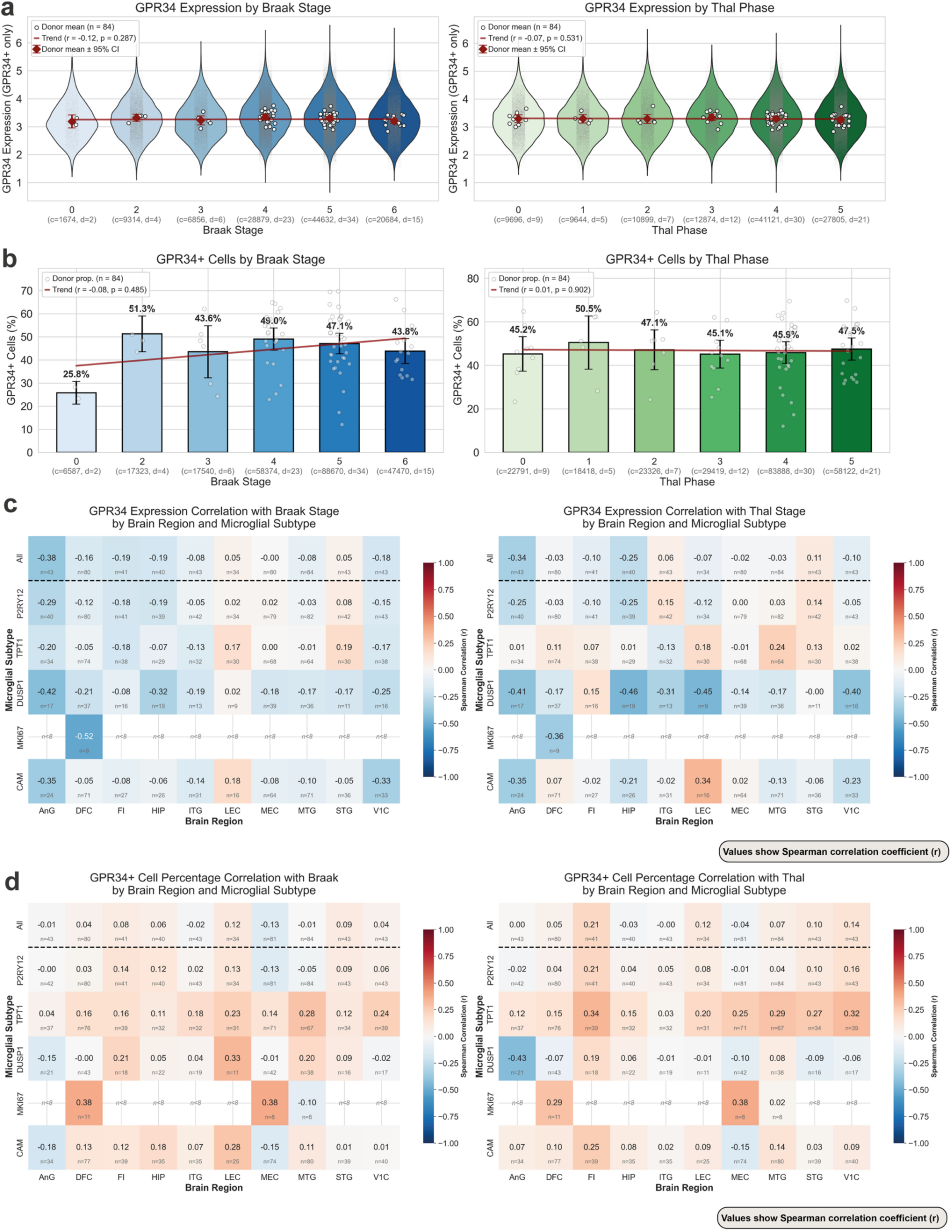
GPR34 expression across Braak stages and Thal phases. **a** Violin plots showing the distribution of GPR34 expression in microglial cells aggregated across all brain regions and microglial subtypes, stratified by Braak stages (left) and Thal phases (right). Individual points represent single nuclei, with sample sizes (c = cells, d = donors) indicated for each stage. Open circles represent per-donor mean GPR34 expression and red diamonds show the donor-level mean ± 95% confidence interval per stage. Red lines and Spearman correlation coefficients (Braak: *r* = − 0.12, *p* = 0.287, Thal: *r* = − 0.07, *p* = 0.531) were computed across donor-level means of GPR34-expressing cells (GPR34+; expression > 0). **b** Bar plots displaying the donor-level proportion of GPR34+ cells for Braak stages (left) and Thal phases (right) across all brain regions and microglial subtypes. Percentages are shown above bars and sample sizes (c = cells, d = donors) below stages. Each open circle is one donor's proportion of GPR34+ microglia; bars show the donor-level mean per stage with 95% confidence intervals. Spearman correlations were computed across donor-level proportions (Braak: *r* = − 0.08, *p* = 0.485; Thal: *r* = +0.01, *p* = 0.902). **c** Heatmaps showing donor-level Spearman correlation coefficients (*r*) between mean GPR34 expression levels and Braak (left) or Thal (right) staging for each brain region (columns) and microglial subtype (rows). Each heatmap cell is one correlation computed across donors, with donor counts annotated as “*n*=”. The top row (“All”) represents correlations across all microglial subtypes combined. “n < 10” indicates region × subtype combinations with too few donors for donor-level analysis. Color scales represent correlation coefficients ranging from *r* = ‒1.0 (negative correlation, blue) to *r* = +1.0 (positive correlation, red). **d** Heatmaps showing donor-level correlation coefficients (r) between the percentage of GPR34+ cells and Braak stages (left) or Thal phases (right) for each brain region and microglial subtype. The percentage of GPR34+ cells was calculated per donor for each pathology stage within each region-subtype combination. Layout and significance indicators are as in panel c. Statistical methods: All inferential tests in this figure are computed at the donor level (*n* = 84 donors; one observation per donor per subgroup). Spearman rank correlation was used to assess monotonic relationships between pathology stages and donor-level GPR34 expression or the percentage of GPR34+ cells. For panels c and d, multiple-testing correction was applied using the Benjamini‒Hochberg FDR method across all region-subtype combinations. Only groups with ≥ 10 nuclei and ≥ 3 valid pathology stages were included in correlation analyses. Region × subtype combinations with fewer than 10 donors were excluded from correlation analyses. Plots were created using Python v3.9

Aggregated donor-level analysis across all brain regions and microglial subtypes revealed no significant variation in GPR34+ cell proportions across Braak stages (Spearman *r* = ‒0.08, *p* = 0.485) or Thal phases (*r* = +0.01, *p* = 0.902; Fig. 11a, b). The absence of an association between GPR34 metrics and pathological staging remained robust after controlling for age using partial Spearman correlations (e.g., age-adjusted correlation for Braak vs. proportion: *r* = ‒0.03, *p* = 0.798; Braak vs. expression: *r* = ‒0.07, *p* = 0.518). Sex-stratified correlations were likewise weak and non-significant for both Braak stage (female: *r* = −0.09, *p* = 0.539; male: *r* = −0.10, *p* = 0.574) and Thal phase (female: *r* = −0.02, *p* = 0.886; male: *r* = +0.05, *p* = 0.768), as were correlations with mean GPR34 expression intensity within each sex (all *p* ≥ 0.231; Supplementary Fig. S4g, h). Formal interaction terms in donor-level linear models revealed no stage-dependent sex effect for either tau (proportion: Braak × sex *β* = +1.05, *p* = 0.643, Supplementary Fig. S4c; expression: *β* = 0.05, *p* = 0.188) or amyloid pathology (proportion: Thal × sex *β* = +1.23, *p* = 0.474, Supplementary Fig. S4d; expression: β = 0.04, p = 0.123).

Region- and subtype-stratified correlation analysis revealed predominantly weak, heterogeneous associations between donor-level GPR34 intensity and pathological staging (Fig. 11c). For Braak stages, point estimates ranged from *r* = ‒0.52 (DFC × MKI67) to *r* = +0.19 (STG × TPT1), with the strongest nominally significant correlation observed in AnG across all microglia (*r* = ‒0.38, *p* = 0.011). For Thal phases, point estimates ranged from *r* = −0.46 (HIP × DUSP1) to *r* = +0.34 (LEC × CAM), with the strongest nominal correlations likewise observed in AnG across all microglia (r = ‒0.33, *p* = 0.027). After Benjamini‒Hochberg FDR correction, no correlation between donor-level mean GPR34 expression and either Braak stage or Thal phase remained significant. The Proportion of GPR34+ microglia showed no significant associations with pathology in any region × subtype combination after FDR correction either (0/102 tests significant, Fig. 11d). Before FDR correction, six region × subtype × stage combinations reached nominal significance (*p* < 0.05). Five of these showed increasing GPR34 with advancing pathology and predominantly involved TPT1 microglia; one combination showed a negative association (AnG × DUSP1 × Thal: *r* = −0.43). None remained significant after correction for multiple testing.

## Discussion

In this study, we investigated microglial changes in human AD brains. Comparative transcriptomic analyses have identified microglia as the brain cell type undergoing the most pronounced changes in response to AD pathology [63, 65, 80, 133]. Our aim was to characterize microglial properties, with a particular focus on GPR34 expression, in relation to Aβ and p-tau deposits, two pathological hallmarks of AD [18]. We analyzed a total of 187,670 microglia in 19 human brains representing Braak stages I−VI, thereby covering a broad spectrum of neuropathological AD progression. The analysis focused on the EC, the earliest site of tau pathology, and the adjacent TC, which becomes affected at later stages [21]. In addition to Braak staging, we quantified the severity of Aβ and p-tau deposits in the EC and TC, as well as the spatial proximity of individual microglia to these deposits. This burden-based and distance-resolved approach was chosen because previous studies and our own data have suggested that the severity of pathological deposits can vary substantially within the same Braak stage (Supplementary Fig. S5; Supplementary Table S5) [85, 119]. It enabled us to investigate interactions between microglia and AD-related deposits in the human brain at the single-cell level. To capture the heterogeneity of microglial responses to AD pathology, we further analyzed 236,002 microglia from an open access snRNA-seq database [44]. This transcriptomic approach allowed us to examine subtype-specific GPR34 expression patterns across microglial states, complementing our spatial analysis with molecular subtype resolution.

### Microglial density, morphology, and GPR34 expression are largely unchanged by AD pathology

Regarding the distribution pattern and cellular properties of microglia, our analyses of both Aβ and p-tau categories revealed a consistent picture: microglial density, morphology (soma size and proximal process length), and GPR34 expression were not significantly altered by the presence or severity of deposits in either the EC or TC. Notably, no regional differences were observed between EC and TC, even in cases where tau pathology was advanced in the EC but absent in the TC. Furthermore, microglia did not accumulate around deposits, and no changes in GPR34 signal counts were detected in the vicinity of Aβ- or AT8-positive deposits. Altogether, our findings contrast with expectations from studies in mice, which predict clustering around deposits, morphological transformation, and transcriptional changes, including regulation of homeostatic genes such as GPR34, if microglia are activated by these deposits [36, 37, 39].

Indeed, we observed a slight, non-significant increase in GPR34 signal counts in cases with high or severe Aβ and p-tau burden (Fig. 4e, f). However, microglia with low and high GPR34 signal counts were frequently found in close spatial proximity (Figs. 5, 6), and distance-resolved analysis revealed no consistent association between GPR34 expression and deposit proximity (Figs. 7a, 8a). Together, these findings argue against a deposit-dependent mechanism underlying the observed category differences. Category comparisons were unadjusted for donor age, PMI, and sex, all three of which correlated with GPR34 signal counts and may therefore introduce residual confounding. Donor age and PMI may affect microglial GPR34 expression in opposite directions [25, 39, 90, 125]. Microglial mRNA has been shown to be remarkably resistant to degradation within the first hours after death, and its levels may even transiently increase depending on the gene [30, 31, 39, 45, 50, 90, 109, 125], whereas transcriptomic studies in both humans and mice have reported age-related declines in the expression of GPR34 and other microglial sensome genes [39, 51, 122]. However, these studies often relied on qPCR for mRNA quantification rather than automated image-based quantification. Sex-specific differences in microglial GPCR expression, including increased GPR34 levels in female microglia, have likewise been described [48, 59, 107]. Notably, in our cohort these variables were interrelated: female donors were on average older and had shorter PMI than male donors (Fig. 9c, f; Supplementary Table S4). In a multivariable analysis including all three variables, none remained independently significant, limiting inference regarding their relative contributions to GPR34 variability. Taken together, the slight increase in GPR34 expression in our study is unlikely to be explained by local Aβ or p-tau depositions alone and may, at least in part, reflect factors that are independent of deposit proximity.

### Distance-dependent microglial changes are subtle

In our distance-resolved analysis, the only deposit-related effect was a shortening of proximal process length in microglia located closer to Aβ deposits, indicating a shift toward less ramified, more amoeboid morphologies (Fig. 7d). This finding is consistent with previous studies on human brain tissue [32, 42]. Importantly, this effect was observed only within a 100 µm radius of plaques and occurred irrespective of overall Aβ burden in the EC and TC. Such subtle, distance-dependent changes would have remained obscured in purely burden-based analyses, underscoring the value of distance-resolved single-cell approaches for uncovering microglial responses to local pathology. Notably, even in immediate plaque proximity, microglia retained a median proximal process length of 10.4 µm, suggesting modest shortening rather than full transformation to an amoeboid phenotype.

Overall, our results demonstrate that microglial properties (density, soma size, proximal process length and GPR34 expression) in the EC and TC remain largely unaffected by Aβ and p-tau deposits, with the only notable changes being a slight, non-significant increase in GPR34 signals in high/severe-burden cases and modest shortening of proximal process length in microglia immediately adjacent to Aβ deposits. The apparent lack of microglial involvement in these pathological processes in human AD is striking, given that numerous mouse studies report pronounced microglial activation at AD-related deposits [27, 43, 63, 113].

### GPR34 expression across subtypes and disease progression

In line with our FISH-based analysis, snRNA-seq revealed no consistent alteration in GPR34 expression in the context of AD. GPR34 expression was preferentially enriched in homeostatic P2RY12 and TPT1 microglia compared to activated DUSP1 microglia (Fig. 10e), consistent with GPR34 being defined as a homeostatic marker whose downregulation accompanies transitions to inflammatory states.

The relationship between GPR34 expression and disease progression showed no consistent pattern. At the donor level, neither the proportion of GPR34+ microglia nor mean GPR34 expression correlated with Braak stage or Thal phase when pooled across regions and subtypes (all |*r*| ≤ 0.12, *p* ≥ 0.287; Fig. 11a, b), and neither measure showed significant heterogeneity across pathological stages (Kruskal‒Wallis *p* ≥ 0.249 for both staging systems). No region × subtype combination survived FDR correction (0/106 for proportion, 0/102 for expression; Fig. 11c, d). Prior to correction, a small number of combinations reached nominal significance, with nominally opposing trends observed: increasing pathology was associated with higher proportions of GPR34+ microglia predominantly in TPT1 across temporal and entorhinal regions (MTG, MEC, and FI), whereas reduced mean expression intensity was observed in other regions (e.g., AnG and HIP). However, none of these associations remained significant after Benjamini‒Hochberg correction, indicating that there is no robust evidence for stage-dependent regulation of GPR34 expression across the SEA-AD cohort. Microglia continuously transition between functional states in response to local microenvironmental cues beyond classical neuropathological hallmarks captured by Braak and Thal staging [15, 33], and GPR34 regulation may therefore reflect aspects of the local tissue environment not resolved by staging alone.

Interestingly, while AD-associated pathology was not a significant predictor of GPR34 expression, donor age showed a modest positive correlation with both the proportion of GPR34+ microglia and mean GPR34 expression in the SEA-AD dataset, consistent with the association between age and GPR34 mRNA signal counts observed in our FISH-based analysis. A similar non-significant trend toward higher GPR34+ proportions in female compared with male donors was also observed in the SEA-AD cohort, independent of Braak or Thal staging and consistent across microglial subtypes and brain regions. Together, these findings across two independent datasets and methodological approaches suggest that baseline GPR34 expression in human microglia is more strongly associated with donor age and sex than with the accumulation of Aβ or p-tau pathology.

Regional differences in baseline GPR34 expression, both in the proportion of GPR34+ microglia (donor-level median 37.7% in HIP vs. 50.7% in V1C) and in expression levels (donor-level median GPR34+ cell expression 3.05 in STG vs. 3.74 in FI), further highlight the influence of local microenvironments on microglial states (Fig. 10d, f). However, these cross-regional differences should be interpreted cautiously, as within-donor analyses restricted to the three regions with the most complete donor overlap (DFC, MEC, MTG) revealed no significant regional variation, suggesting that part of the apparent heterogeneity may reflect differences in donor composition across regions rather than intrinsic regional effects. Taken together, these findings indicate that GPR34 expression in human microglia is multifactorial and context-dependent, shaped by microglial subtype identity, regional environment, and donor demographics, with no robust evidence for systematic stage-dependent modulation in either cohort examined.

### Reconciling human and mouse findings

Our analyses reveal complex, subtype-specific regulation of GPR34 during AD, providing a framework to reconcile conflicting literature reports. While some studies report GPR34 upregulation in AD [100, 132], others emphasize downregulation [63]. These divergent findings likely reflect differences in sampled microglial populations or brain regions rather than direct effects of AD pathology on GPR34 expression.

Consistent with previous studies, human microglial transcriptional states do not fully recapitulate those seen in murine models [1, 76, 77]. Human microglia are more heterogeneous and context-dependent [76–78, 89, 95], as evident in our FISH-IF analyses, where microglia within the same case displayed substantial variability in GPR34 expression and morphology. In Aβ-depositing mouse strains (e.g., APP/PS1 and Nlgn4x), microglia exhibit strong GPR34 upregulation and amoeboid morphology [39, 52, 71, 93]. By contrast, in human tissue, microglial morphology remained largely unchanged, with only subtle activation and highly variable GPR34 expression, consistent with several human transcriptomic studies [39, 47, 82].

Potential explanations for species differences include (i) artificial replication of AD-associated changes in transgenic mice may have unintended effects, including ectopic gene integration, neurotoxicity from expression systems, and artificial overexpression/aggregation of foreign proteins [126]. Human Aβ and tau aggregation mechanisms remain poorly understood, complicating replication in mice [87, 131]. (ii) Mouse models are typically studied at 3‒12 months, corresponding to < 50 years in humans [40, 46]. Aging microglia exhibit dystrophy, reduced phagocytic activity, and increased pro-inflammatory signaling, potentially affecting GPR34 expression [11, 29, 32, 36, 39, 68, 97]. (iii) Mouse microglia encounter pathology throughout life, including during development, while human microglia face AD pathology primarily in late life, except in rare genetic forms [23, 88]. (iv) Mouse models often fail to recapitulate the full spectrum and regional pattern of human AD pathology [36]. (v) Species-specific differences in GPR34 regulation exist [6, 38] and expression may differ depending on whether microglia encounter Aβ or p-tau deposits [39, 98].

### Post-mortem considerations and methodological limitations

Microglia were identified using the pan-microglial marker Iba1, which labels both resting and activated microglia, including their cellular processes [54, 101, 130]. All Iba1-positive cells were manually reviewed to minimize potential misclassification. However, a small subset of Iba1-negative microglia may have escaped detection [62, 118], and a minor contribution of non-microglial Iba1-positive myeloid cells cannot be entirely excluded [54, 55, 121], as peripheral myeloid cells have been described to enter the CNS and acquire microglia-like morphology in neurodegenerative conditions (e.g., axonal damage) [13, 68, 83]. Notably, a subset of disease-associated microglial states has been shown to downregulate Iba1 expression in AD and may therefore not be fully captured by our analysis [68, 118].

It could be argued that deposit-induced changes in GPR34 or morphology were masked by post-mortem microglial activation (e.g., due to oxygen deprivation or neuronal death) [31, 109]. However, prior studies indicate that microglial morphology and gene expression remain largely stable for 12−24 h post-mortem, with only minor transcriptional changes [45, 50, 109, 120]. Beyond this interval, post-mortem effects may increase but would likely affect all microglia uniformly within a sample. Consequently, masking of pathology-associated changes at the intra-individual level is unlikely, although inter-individual PMI variability remains a potential bias. Additionally, GPR34 expression may be influenced by donor sex, pre-mortem conditions, brain region, gray-white matter composition, tissue handling, and methodological approach [12, 35, 50, 61, 96, 105, 122], which should be considered when comparing studies. Both datasets analyzed here were limited to cortical and hippocampal grey matter; GPR34 expression in white matter microglia remains to be characterized.

A central limitation of the snRNA-seq analysis is the substantial inter-individual heterogeneity of microglial states observed across donors (Figs. 10e, f; 11a, b). Within the same microglial subtype, per-donor GPR34+ proportions vary by approximately 2–3-fold, exceeding the variation observed across pathology stages. This variability likely reflects unmeasured factors influencing microglial transcriptional state and cannot be resolved using a cross-sectional design that provides only a single transcriptional snapshot per donor. Longitudinal or spatially resolved approaches would be required to capture dynamic regulation of GPR34 during disease progression within individuals.

A further limitation is that our analyses were restricted to transcript-level measurements. Although protein-level validation of GPR34 would be informative, reliable detection of endogenous human GPCRs in native tissue is often limited by the lack of sufficiently validated antibodies [57, 58, 74, 81]. In situ hybridization and single-cell or single-nucleus RNA sequencing therefore remain among the most reliable approaches currently available for assessing endogenous GPR34 expression in human brain tissue. Furthermore, GPCR-mediated signal transduction can vary independently of receptor abundance due to differences in ligand availability, receptor coupling efficiency, and intracellular signaling context [39, 104, 107]. Accordingly, the absence of changes in GPR34 mRNA levels does not exclude functionally relevant alterations in GPR34 signaling. Lysophospholipids released during demyelination and synaptic damage have been shown to activate GPR34 on microglia and promote pro-inflammatory cytokine release in AD [70, 92].

### Implications and future directions

Our study adds new insights into the complex and often debated role of microglia in AD [66]. However, the cross-sectional nature of snRNA-seq and post-mortem analyses limits the ability to resolve dynamic GPR34 changes within individuals over time. Longitudinal studies and functional validation in human tissue or organoid models will be required to clarify whether GPR34 plays a causal role in AD pathogenesis.

Overall, our results highlight the complex interplay between aging, microglial heterogeneity, and methodological variation underlying divergent patterns of GPR34 expression. In the brains of elderly individuals examined here, GPR34-expressing microglia appeared to play only a minor role in relation to Aβ and p-tau pathology. This raises the question of whether the limited response reflects age-related functional decline, or an intrinsic feature of microglia in the human AD brain [114, 116]. If age-related decline predominates, further questions arise: do aged microglia fail to recognize AD pathology? Does the disease alter their sensory or signaling capacities in ways not yet understood? Could therapeutic modulation of aged microglia, perhaps via GPR34, be protective—or might it exacerbate disease progression [39]?

In sum, microglia are central to AD, yet their precise role in the human brain—whether protective, detrimental, or neutral—remains a critical mystery.

## Supplementary Information

Below is the link to the electronic supplementary material.

Supplementary file1 (PDF 1411 kb)

## Data Availability

All data and materials generated in this study, including microscopy images and analytical outputs, are available from the corresponding author upon reasonable request. The snRNA-seq data were obtained from the Seattle Alzheimer's Disease Brain Cell Atlas (SEA-AD) consortium and are publicly available through the Allen Institute Brain Map data portal (https://brain-map.org/consortia/sea-ad/our-data). The microglia and immune cell dataset is accessible through the SEA-AD data repository at https://sea-ad-single-cell-profiling.s3.amazonaws.com/index.html#Microglia-and-Immune-for-AAIC/. Custom analysis scripts used for data processing and visualization are available from the corresponding author upon reasonable request.
