# Supplementary material for: Expression of GPR34 in microglia remains stable in human Alzheimer’s disease
Source: Acta Neuropathol. 2026 Jun 15;151(1):66. doi: 10.1007/s00401-026-03035-0 (PMC13269318; doi:10.1007/s00401-026-03035-0)
Supplement: Supplementary file 1 — Supplementary file1 (PDF 1411 kb) [file 401_2026_3035_MOESM1_ESM.pdf]

Supplementary Figures

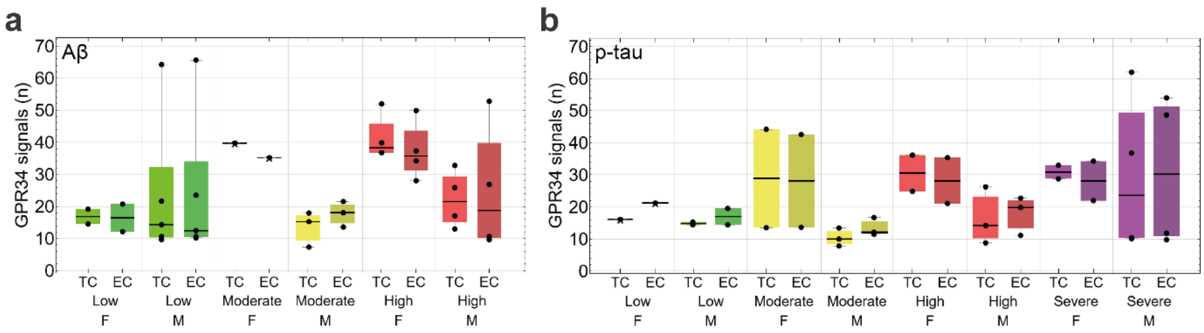

Figure S1 Sex differences in GPR34 mRNA signal counts across Aβ and p-tau burden categories

a & b: GPR34 mRNA signal counts per Iba1-positive microglia in the temporal cortex (TC) and entorhinal cortex (EC), stratified by sex (F: female, M: male) within each Aβ burden category (a) and p-tau burden category (b). Colors indicate pathology burden categories as defined in the Material & Methods (low: green, moderate: yellow, high: red, severe: purple). Each dot represents the median value per case, calculated across all microglia analyzed within the respective region. Boxes indicate the interquartile range (IQR) with the median line; whiskers extend to 1.5× IQR; points beyond whiskers represent outliers. Female donors showed a trend toward higher GPR34 mRNA signal counts per microglia across all brain regions and pathology categories compared with male donors, although this difference did not reach statistical significance. Plots were generated with Wolfram Mathematica v14.0.

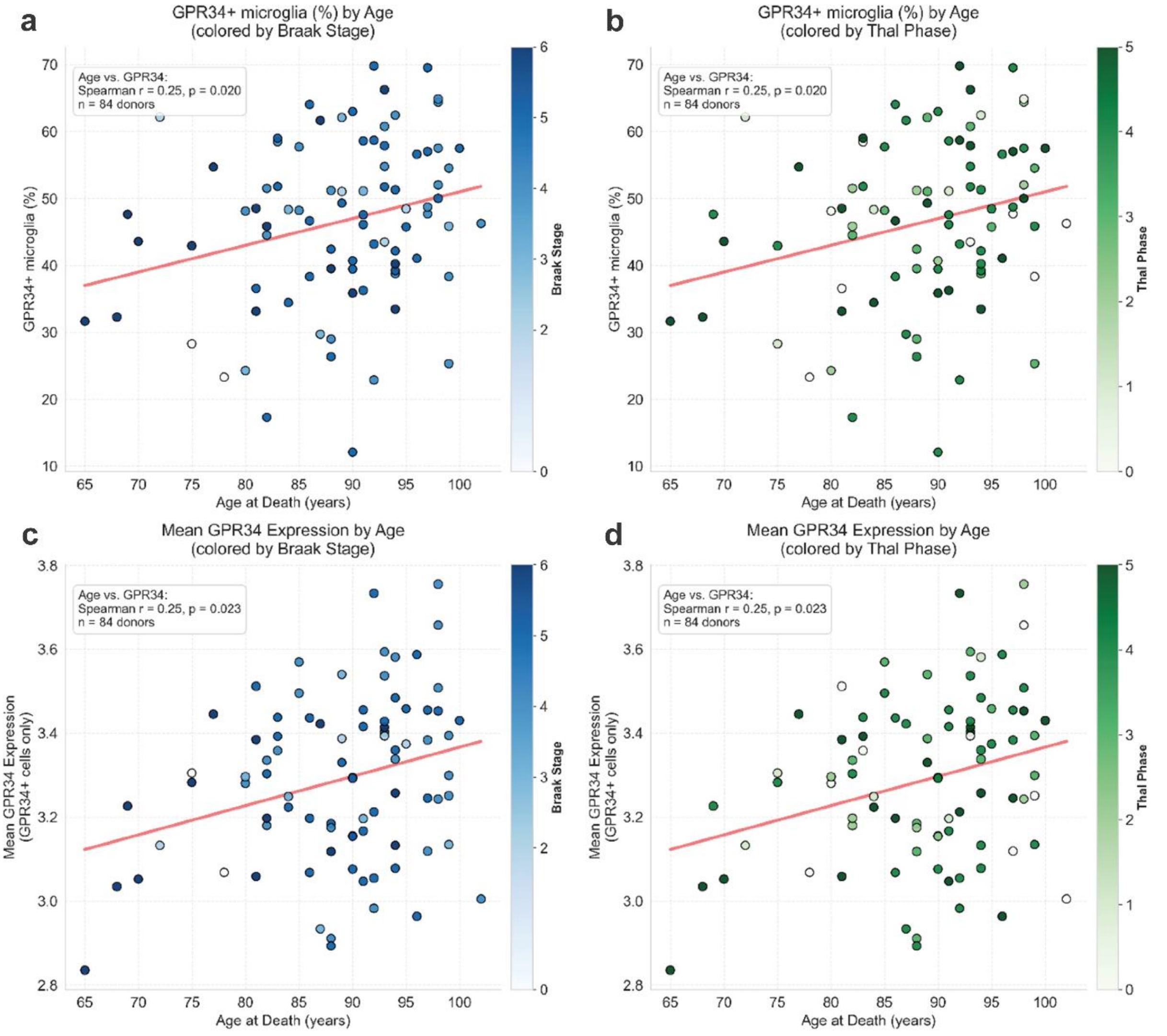

Figure S2 Correlation between donor age and microglial GPR34 expression across Braak stages and Thal phases Donor-level analysis of the SEA-AD dataset. Scatter plots show the relationship between age at death (in years) and donor-level GPR34 metrics. Each data point represents a summary statistic for an individual donor, computed from all microglia analyzed per donor. Data points are color-coded by Braak stage (panels a and c; blue scale) or Thal amyloid phase (panels b and d; green scale) to illustrate the distribution of pathology across age. **a & b**: Relationship between age and the proportion of GPR34-positive microglia per donor. Across all donors, a moderate but significant positive correlation was observed (Spearman r = 0.25, p = 0.020). **c & d**: Relationship between age and mean GPR34 expression among GPR34-positive cells. This analysis similarly shows a significant positive association (Spearman r = 0.25, p = 0.023). Solid red lines represent ordinary least squares regression fits. Statistical associations between continuous demographic variables and GPR34 metrics were assessed using two-sided Spearman rank correlation. Plots were generated using Python v3.9.

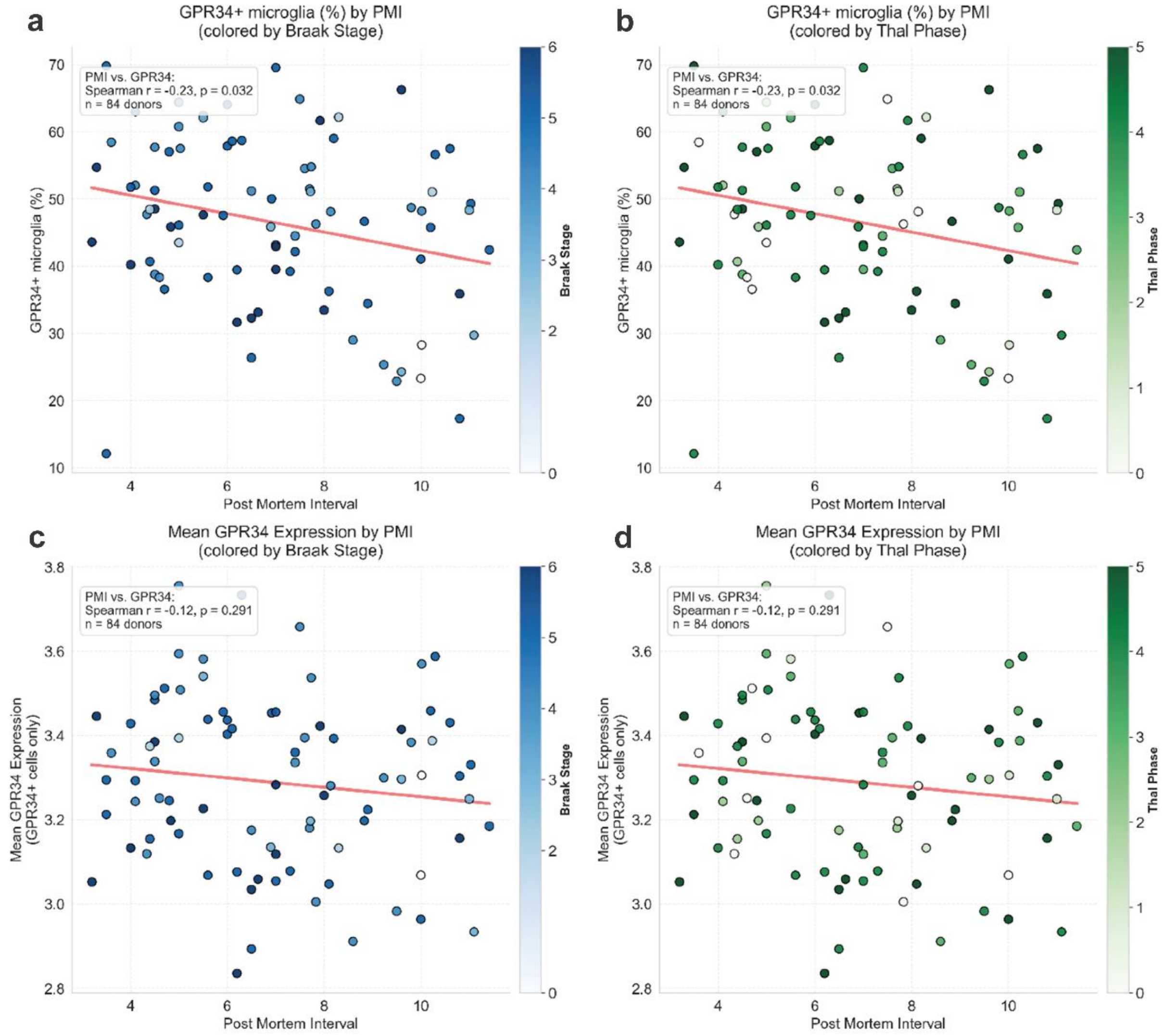

Figure S3 Correlation between the postmortem interval (PMI) and microglial GPR34 expression across Braak stages and Thal phases

Donor-level analysis of the SEA-AD dataset. Scatter plots show the relationship between the PMI and donor-level GPR34 metrics. Data points are color-coded by Braak stage (panels a and c; blue scale) or Thal amyloid phase (panels b and d; green scale). **a & b**: Relationship between PMI and the proportion of GPR34-positive microglia per donor. A significant negative correlation was observed (Spearman r = –0.23, p = 0.032). **c & d**: Relationship between PMI and mean GPR34 expression intensity among GPR34-positive cells. This association did not reach statistical significance (r = –0.12, p = 0.291). Solid red lines represent ordinary least squares regression fits. Statistical associations between continuous technical variables and GPR34 metrics were assessed using two-sided Spearman rank correlation. Plots were generated using Python v3.9.

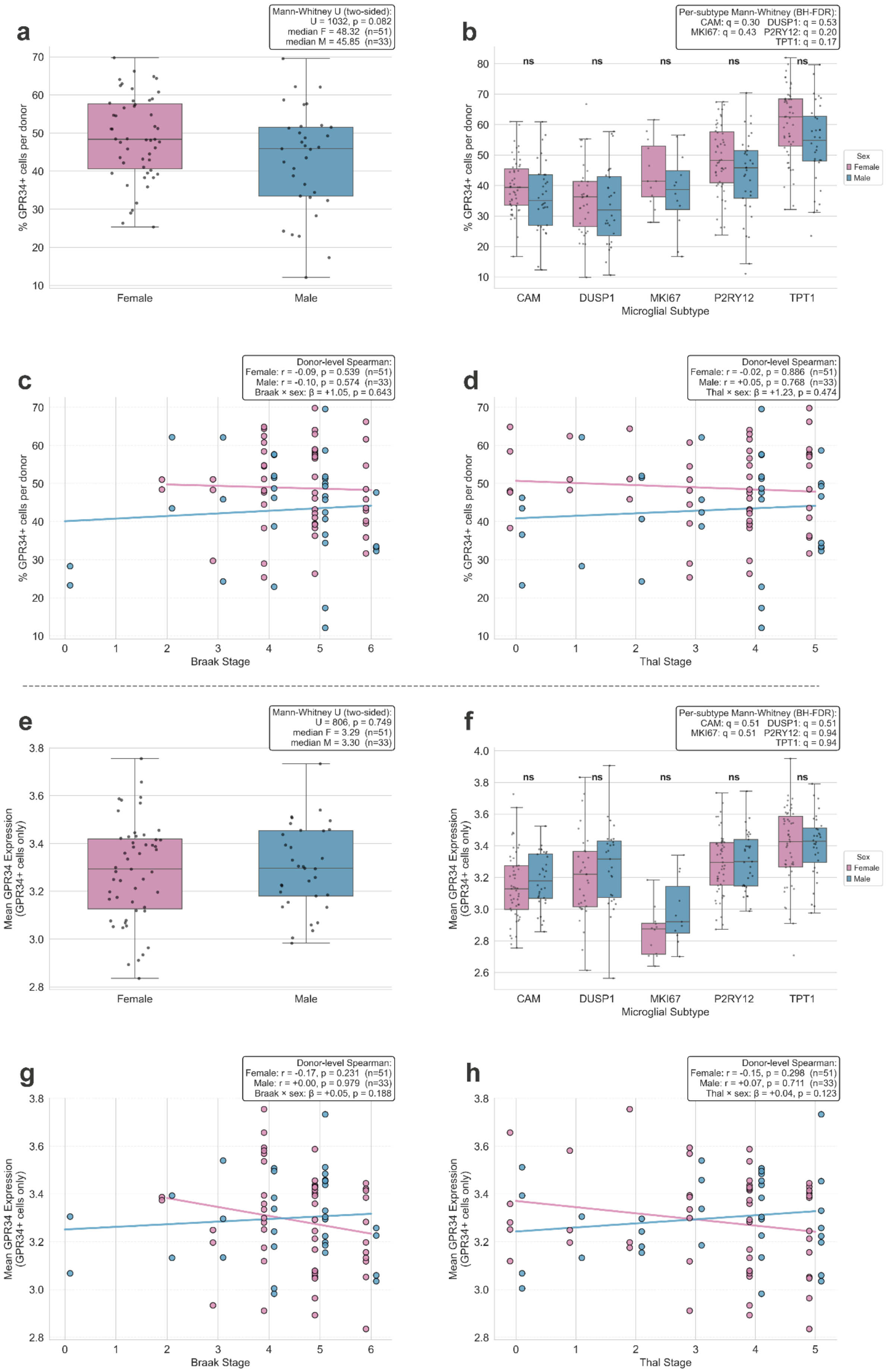
a
Mann-Whitney U (two-sided):
U = 1032, p = 0.082
median F = 48.32 (n=51)
median M = 45.85 (n=33)
% GPR34+ cells per donor
Female
Male
b
Per-subtype Mann-Whitney (BH-FDR):
CAM: q = 0.30 DUSP1: q = 0.53
MKI67: q = 0.43 P2RY12: q = 0.20
TPT1: q = 0.17
ns
% GPR34+ cells per donor
Sex
Female
Male
CAM
DUSP1
MKI67
P2RY12
TPT1
Microglial Subtype
c
Donor-level Spearman:
Female: r = -0.09, p = 0.539 (n=51)
Male: r = -0.10, p = 0.574 (n=33)
Braak × sex: β = +1.05, p = 0.643
% GPR34+ cells per donor
Braak Stage
d
Donor-level Spearman:
Female: r = -0.02, p = 0.886 (n=51)
Male: r = +0.05, p = 0.768 (n=33)
Thal × sex: β = +1.23, p = 0.474
% GPR34+ cells per donor
Thal Stage
e
Mann-Whitney U (two-sided):
U = 806, p = 0.749
median F = 3.29 (n=51)
median M = 3.30 (n=33)
Mean GPR34 Expression
(GPR34+ cells only)
Female
Male
f
Per-subtype Mann-Whitney (BH-FDR):
CAM: q = 0.51 DUSP1: q = 0.51
MKI67: q = 0.51 P2RY12: q = 0.94
TPT1: q = 0.94
ns
Mean GPR34 Expression
(GPR34+ cells only)
Sex
Female
Male
CAM
DUSP1
MKI67
P2RY12
TPT1
Microglial Subtype
g
Donor-level Spearman:
Female: r = -0.17, p = 0.231 (n=51)
Male: r = +0.00, p = 0.979 (n=33)
Braak × sex: β = +0.05, p = 0.188
Mean GPR34 Expression
(GPR34+ cells only)
Braak Stage
h
Donor-level Spearman:
Female: r = -0.15, p = 0.298 (n=51)
Male: r = +0.07, p = 0.711 (n=33)
Thal × sex: β = +0.04, p = 0.123
Mean GPR34 Expression
(GPR34+ cells only)
Thal Stage

Figure S4 Sex-specific differences in microglial GPR34 expression across Braak stages and Thal phases

Donor-level analysis of the SEA-AD dataset (84 donors: 51 female, 33 male). Each data point represents one donor, summarized across all microglia sampled from that donor. Female donors are shown in pink and male donors in blue throughout. **a–d:** Proportion of GPR34-expressing (GPR34+) microglia per donor. **a & b**: Box plots showing the proportion of GPR34+ microglia by sex (a) and stritified by microglial subtype (CAM, DUSP1, MKI67, P2RY12, TPT1) and sex (b). Donor × subtype strata containing fewer than 20 cells were excluded. Boxes indicate the interquartile range with the median line. No significant sex differences were observed overall (Mann−Whitney U = 1032, p = 0.082) or within any subtype (all FDR-adjusted q ≥ 0.16). **c & d:** Donor-level proportion of GPR34+ microglia plotted against Braak stage (c) and Thal phase (d), stratified by sex. Per-sex Spearman correlations were weak and non-significant for Braak stage (female: r = −0.09, p = 0.539; male: r = −0.10, p = 0.574) and Thal phase (female: r = −0.02, p = 0.886; male: r = 0.05, p = 0.767). Braak × sex (β = +1.05, p = 0.643) and Thal × sex (β = +1.23, p = 0.473) interaction terms were likewise non-significant. **e–h:** Mean GPR34 expression intensity among GPR34+ microglia per donor. **e & f:** Box plots showing mean GPR34 expression by sex (e) and stratified by microglial subtype and sex (f). No significant sex differences were observed overall (Mann−Whitney U = 806, p = 0.749) or within any subtype (all FDR-adjusted q ≥ 0.51). **g & h:** Donor-level mean GPR34 expression plotted against Braak stage (g) and Thal phase (h), stratified by sex. Per-sex Spearman correlations were non-significant for Braak stage (female: r = −0.17, p = 0.230; male: r = 0.00, p = 0.979) and Thal phase (female: r = −0.15, p = 0.298; male: r = 0.07, p = 0.711). Braak × sex (β = +0.05, p = 0.188) and Thal × sex (β = +0.05, p = 0.123) interaction terms were non-significant. Solid lines in **c**, **d**, **g**, and **h** show ordinary least squares fits per sex. Statistical methods: In panels **a**, **b**, **e**, and **f**, groups were compared using two-sided Mann−Whitney U tests; in panels **b** and **f**, multiple-testing correction across subtypes was performed using the Benjamini–Hochberg (FDR) procedure. In panels **c**, **d**, **g**, and **h**, correlations were assessed using per-sex Spearman rank correlation, and stage × sex interactions were tested using donor-level linear regression models including pathology stage, sex, and their interaction. Plots were generated using Python v3.9.

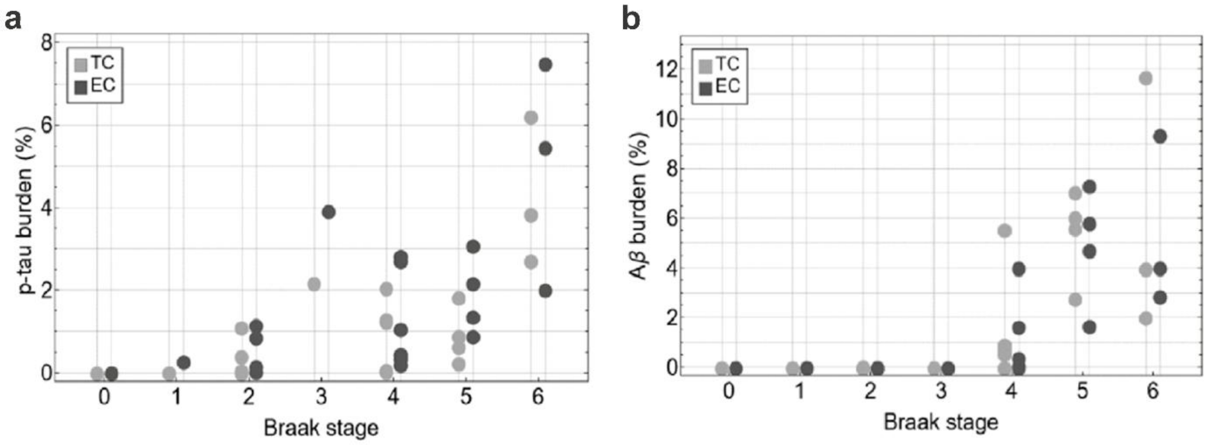

Figure S5 Inter-individual variability of p-tau and Aβ burden across Braak stages

a & b: Regional p-tau (a) and Aβ (b) burden in the temporal cortex (TC, light gray) and entorhinal cortex (EC, dark gray) across Braak stages. Each dot represents an individual case. P-tau and Aβ burdens were quantified as the percentage of tissue area occupied by p-tau or Aβ deposits. Regional p-tau and Aβ burden varied considerably between cases within the same Braak stage, particularly at advanced stages (Braak IV–VI). Plots were generated using Wolfram Mathematica v14.0.

Supplementary Tables

Table S1: Image acquisition parameters

PARAMETER	VALUE/SETTING
Scanner model	Axioscan 7 (Zeiss Group, Jena, Germany)
Scanner software	ZEN 3.7
**Fluorescence imaging**	
Objective magnification	40 ×
Pixel size	0.172 µm
Z-stack layers	11
Inter-slice distance	0.5 µm
Fluorescence channels	DAPI, AF647, AF488, Cy3
EDF method	Variance
EDF contrast lenght scale	3
EDF smoothing	1
EDF reconstruction threshold	0.05
**Brightfield imaging**	
Objective magnification	20 ×
Pixel size	0.344 µm

DAPI = 4',6-diamidino-2-phenylindole, **AF647** = Alexa Fluor 647, **AF488** = Alexa Fluor 488, **EDF** = extended depth of focus

Table S2: Cell detection and classification parameters

PARAMETER	VALUE/SETTING	PURPOSE/NOTES
Analysis platform	QuPath 0.5.1	Whole-slide image analysis
Cell detection algorithm	StarDist 0.5.0	Deep learning-based nucleus detection
**Nucleus detection**		
Channel	DAPI	
Normalization	Percentile [1, 99]	Contrast adjustment prior to detection
Detection threshold	0.25	Minimum normalized intensity for nuclei
Pixel size factor	0.9	Scaling factor for detection sensitivity
Output measurement	Intensity	Mean, median, SD per nucleus
**Microglia selection**		
Channel	AF647	
Median intensity threshold	>375	Minimum Iba1 signal intensity
SD threshold	>100	Excludes uniform low-intensity background
Nucleus–Iba1 overlap criterion	≥60% AF647-positive nucleus area	Ensures spatial co-localization of nucleus and cytoplasm
**Exclusion of false positives**		
Channel	AF488	
Median intensity threshold	<1000	Removal of non-specific signals
**Object classifier training**		
Feature set	AF647 intensity (mean, median, min, max, SD)	Captures signal distribution and spatial heterogeneity
	AF488 intensity (mean, median, min, max, SD	
	AF647 Haralick texture features (F5, F10)	
Training set (positive class)	29,435 detections	Manually validated microglia
Training set (negative class)	591,153 detections	non-microglial detections (e.g. artifacts, other cell types)

DAPI = 4',6-diamidino-2-phenylindole, **AF647** = Alexa Fluor 647, **AF488** = Alexa Fluor 488, **SD** = standard deviation, **F3** = Haralick texture feature 3, **F10** = Haralick texture feature 10

Table S3: Image processing, morphological segmentation, and quantification parameters

PARAMETER	VALUE/SETTING	PURPOSE/NOTES
Software	Mathematica 14.0	Image processing and quantification
Input format	401×401 px tiles (4-channel TIFF)	Centered on individual microglia
**Microglial soma segmentation**		
Channel	AF647	
Thresholding	Otsu method	Automatic global segmentation
Morphological operations	5 px closing, hole filling, 5 px opening	Smooths contours, removes artifacts
AF488 subtraction mask threshold	0.0025	Removal of non-microglial structures
Output	***Soma size (μm²)***	
**Microglial process segmentation**		
Channel	AF647	
Preprocessing	3 px ridge filter	Removal of non-specific signals
Morphological closing	1 px	Connects fragmented segments
Process assignment	Within 75 px of soma center	Links processes to parent cell
Skeletonization	1 px medial axis	Enables length measurement
Output	***Proximal process length (μm)***	
**Microglial density quantification**		
Radius	150 μm	Reflects immediate cellular microenvironment
Output	***Local microglial density (n within 150 μm)***	
ROI level	EC, TC	Overall microglial abundance per region
Output	***Regional microglial density (n/mm²)***	
**GPR34 mRNA quantification**		
Channel	Cy3	
Segmentation	Local adaptive thresholding	Detects individual signal dots
Average single-dot intensity	0.66	Normalization factor for transcript quantification
Size exclusion	<5 px (0.15 μm²)	Removes noise
Quantification	Cumulative intensity / single-dot intensity	Estimates transcript count (manufacturer protocol)
Output	***GPR34 signal count (n)***	

TIFF = tagged image file format, **px** = pixel, **AF647** = Alexa Fluor 647, **AF488** = Alexa Fluor 488, **ROI** = region of interest, **EC** = entorhinal cortex, **TC** = temporal cortex, **GPR34** = G protein-coupled receptor 34

Table S4: Donor characteristics and microglial parameters in male and female donors across Aβ and p-tau burden categories

Aβ BURDEN		LOW	MODERATE	HIGH
AGE (y)	F	**70.0** (32.0)	**80.0** (0.0)	**86.5** (6.0)
	M	**71.0** (15.0)	**83.0** (11.3)	**79.5** (9.5)
PMI (h)	F	**41.0** (14.0)	**32.0** (0.0)	**22.0** (23.0)
	M	**48.0** (50.5)	**48.0** (18.0)	**18.5** (28.5)
Aβ BURDEN (%)	F	**0.0003** (0.0004)	**0.4164** (0.0000)	**4.9470** (3.2806)
	M	**0.0007** (0.0014)	**0.4151** (0.8745)	**4.9796** (4.0901)
MG DENSITY (n/mm²)	F	**46.81** (6.30)	**93.86** (0.00)	**76.57** (25.06)
	M	**77.00** (15.95)	**88.99** (24.55)	**58.25** (73.18)
GPR34 SIGNAL COUNT (n)	F	**16.45** (6.75)	**36.01** (0.00)	**36.42** (9.97)
	M	**13.10** (22.50)	**16.75** (6.44)	**18.95** (25.29)
MG SOMA SIZE (µm²)	F	**109.50** (4.64)	**86.78** (0.00)	**99.61** (25.57)
	M	**99.24** (16.28)	**115.60** (29.35)	**104.40** (23.57)
MG PROCESS LENGTH (µm)	F	**12.45** (7.89)	**18.91** (0.00)	**13.01** (5.68)
	M	**12.02** (2.94)	**7.90** (4.09)	**11.48** (4.55)
Aβ DEPOSIT DISTANCE (µm)	F	**7,138.00** (4,079.00)	**387.30** (0.00)	**56.33** (12.86)
	M	**9,044.00** (10,440.00)	**1,053.00** (896.20)	**71.32** (44.11)

P-TAU BURDEN		LOW	MODERATE	HIGH	SEVERE
AGE (y)	F	**86.0** (0.0)	**70.0** (32.0)	**83.0** (6.0)	**92.0** (10.0)
	M	**63.5** (17.0)	**71.0** (1.5)	**81.0** (18.0)	**84.5** (6.5)
PMI (h)	F	**34.0** (0.0)	**50.0** (4.0)	**24.5** (15.0)	**21.5** (11.0)
	M	**60.0** (24.0)	**72.0** (0.0)	**24.0** (24.0)	**18.5** (17.5)
P-TAU BURDEN (%)	F	**0.1374** (0.0000)	**0.9434** (0.0608)	**1.4073** (0.6403)	**3.6275** (2.4738)
	M	**0.0658** (0.1164)	**0.2387** (0.0478)	**1.0075** (1.1210)	**2.8534** (2.5530)
MG DENSITY (n/mm²)	F	**42.46** (0.00)	**45.83** (52.29)	**59.12** (25.57)	**57.50** (35.53)
	M	**89.29** (21.56)	**64.37** (31.05)	**76.61** (37.04)	**114.60** (76.17)
GPR34 COUNT (n)	F	**18.59** (0.00)	**28.36** (29.55)	**28.63** (13.94)	**29.38** (9.02)
	M	**15.50** (2.15)	**12.59** (3.01)	**17.19** (11.96)	**28.10** (40.56)
MG SOMA SIZE (µm²)	F	**107.70** (0.00)	**98.69** (20.93)	**104.30** (21.92)	**96.95** (16.91)
	M	**83.53** (8.45)	**98.99** (7.05)	**94.50** (8.66)	**92.33** (20.81)
MG PROCESS LENGTH (µm)	F	**14.50** (0.00)	**11.46** (19.20)	**7.00** (7.99)	**9.22** (0.43)
	M	**8.55** (3.71)	**11.85** (2.43)	**6.89** (0.59)	**12.22** (6.17)
P-TAU DEPOSIT DISTANCE (µm)	F	**51.62** (0.00)	**20.57** (3.38)	**12.28** (1.19)	**6.65** (4.15)
	M	**151.00** (163.20)	**52.61** (13.47)	**16.79** (12.63)	**6.84** (2.16)

Median values with interquartile ranges (in parentheses) are given. **F** = female, **M** = male, **AGE (y)** = age at death in years (y), **PMI (h)** = post-mortem interval in hours (h), **Aβ BURDEN (%)** = percentage of tissue area occupied by amyloid-β, **P-TAU BURDEN (%)** = percentage of tissue area occupied by hyperphosphorylated tau, **MG DENSITY (n/mm²)** = regional density of microglia in cells per mm², **GPR34 SIGNAL COUNT (n)** = number of GPR34 mRNA signals per cell, **MG SOMA SIZE (µm²)** = microglial soma area in µm², **MG PROCESS LENGTH (µm)** = microglial proximal process length in µm, **Aβ DEPOSIT DISTANCE (µm)** = median distance of each microglia to the nearest Aβ deposit, **P-TAU DEPOSIT DISTANCE (µm)** = median distance of each microglia to the nearest p-tau deposit

Table S5: Quantitative regional p-tau and Aβ burden used for case categorization

CASE ID	P-TAU CATEGORY	Aβ CATEGORY	BRAAK	TC P-TAU BURDEN (%)	EC P-TAU BURDEN (%)	TC Aβ BURDEN (%)	EC Aβ BURDEN (%)
1	1	1	0	0.006	0.010	0.000	0.000
2	1	1	2	0.061	0.160	0.000	0.000
3	1	1	4	0.073	0.206	0.001	0.000
4	2	1	1	0.009	0.278	0.002	0.003
5	2	1	2	0.007	0.033	0.029	0.003
6	2	1	2	0.404	1.161	0.000	0.000
7	2	2	4	0.033	0.352	0.552	0.053
8	2	3	4	0.051	0.459	5.548	4.008
9	2	3	5	0.235	1.368	7.059	7.315
10	3	1	2	1.110	0.865	0.000	0.002
11	3	2	4	1.303	2.836	0.913	0.082
12	3	2	4	1.240	1.069	0.671	0.377
13	3	3	5	0.638	0.893	2.771	1.669
14	3	3	5	0.894	2.173	6.036	5.819
15	4	1	3	2.181	3.924	0.000	0.002
16	4	2	4	2.060	2.722	0.893	1.639
17	4	3	5	1.834	3.089	5.603	4.722
18	4	3	6	2.722	2.017	3.977	4.015
19	4	3	6	6.213	7.498	11.697	9.349
20	4	3	6	3.846	5.463	2.016	2.861

CASE ID = case Identification number, **P-TAU CATEGORIES** (1–4) correspond to low, moderate, high, and severe p-tau burden, **Aβ CATEGORIES** (1–3) correspond to low, moderate, and high Aβ burden, **BRAAK** = Braak stage, **Aβ BURDEN (%)** = percentage of tissue area occupied by amyloid-β relative to the total tissue area in the temporal cortex (**TC**) and entorhinal cortex (**EC**)**, P-TAU BURDEN (%)** = percentage of tissue area occupied by hyperphosphorylated tau relative to the total tissue area in the temporal cortex (**TC**) and entorhinal cortex (**EC**)
